# Computational Study on the Pd-Catalyzed Pathway for the Formation of (*R*)-Methyl-(2-Hydroxy-1-Phenylethyl)Carbamate

**DOI:** 10.3390/molecules30081781

**Published:** 2025-04-16

**Authors:** Silvia González, Consuelo Mendoza Herrera, Lydia María Pérez Díaz, Laura Orea Flores, José Antonio Rivera Márquez, Ximena Jaramillo-Fierro

**Affiliations:** 1 Departamento de Química, Facultad de Ciencias Exactas y Naturales, Universidad Técnica Particular de Loja, París s/n y Praga, Loja 110107, Ecuador; xvjaramillo@utpl.edu.ec; 2 Decanato de Ciencias de la Vida y la Salud, Universidad Popular Autónoma del Estado de Puebla, 21 Sur 1103, Bario de Santiago, Puebla 72410, Mexico; 3 Facultad de Ingeniería Química, BUAP, C.U., Edif. 106H, Blvd. 18 Sur y Av. San Claudio, Col. San Manuel, Puebla 72570, Mexico; maria.mendozah@correo.buap.mx (C.M.H.); lydia_perez@live.com.mx (L.M.P.D.); laura.orea@correo.buap.mx (L.O.F.); jose.rivera@correo.buap.mx (J.A.R.M.)

**Keywords:** carbamate, palladium, catalysis, reaction pathway, DFT study, HPC, NMR

## Abstract

The formation of (*R*)-methyl-(2-hydroxy-1-phenylethyl)carbamate through Pd(PPh_3_)_4_-catalyzed synthesis was investigated using computational methods to elucidate the reaction pathway and energetic feasibility. Density functional theory (DFT) calculations confirmed that the direct reaction between (*R*)-(-)-2-phenylglycinol and methyl chloroformate is not spontaneous, requiring a catalyst to proceed efficiently. The study proposes a detailed mechanistic pathway involving ligand dissociation, intermediate formation, and hydrogenation. The role of Pd(PPh_3_)_4_ was examined, demonstrating its ability to stabilize reaction intermediates and facilitate key transformations, such as dehydrogenation and chlorine elimination. Two reaction pathways were identified, with Pathway 1 exhibiting a net energy of –84.7 kcal/mol and Pathway 2 showing an initial positive energy of 90.1 kcal/mol. However, the regeneration of key intermediates in Pathway 2 ultimately reduces the total reaction energy to –238.7 kcal/mol, confirming the feasibility of both routes. Computational results align with experimental NMR data, supporting the formation of the proposed intermediates. These findings provide valuable insights into catalyst optimization, suggesting that ligand modifications or alternative palladium-based catalysts could enhance efficiency. This study advances the understanding of Pd-catalyzed carbamate synthesis and offers a basis for future experimental and computational investigations.

## 1. Introduction

Carbamates are an essential class of organic compounds characterized by the -C(=O)O-N- functional group, which grants them unique physicochemical properties, including high chemical stability and tunable reactivity [1]. Due to their versatility, carbamates have been widely studied and applied in various fields, including pharmaceuticals, agriculture, and materials science [2,3,4]. In the pharmaceutical industry, carbamates are key components in drug delivery systems and are found in various bioactive molecules with pharmacological relevance [5]. These compounds exhibit a broad range of biological activities, including acetylcholinesterase inhibitor, anticancer, antimicrobial, and anti-inflammatory effects, making them attractive scaffolds for medicinal chemistry [6,7]. For instance, Rivastigmine, a carbamate-based drug, is an acetylcholinesterase inhibitor widely used for treating Alzheimer’s disease, providing symptomatic relief by enhancing cholinergic function. Additionally, research continues to develop multifunctional carbamate derivatives that target various pathological aspects of Alzheimer’s disease, including oxidative stress and amyloid-beta aggregation [8,9,10]. Beyond their pharmaceutical applications, carbamates are also widely employed in the agrochemical sector, where they serve as active ingredients in pesticides, fungicides, and herbicides [11]. Their ability to inhibit acetylcholinesterase in insects has made them effective agents for pest control, although concerns regarding their environmental persistence and potential toxicity have prompted research into alternative synthesis methods [12]. Moreover, carbamates are increasingly being explored for their role in polymer chemistry, where they contribute to the development of sequence-defined polymers with enhanced structural rigidity and tailored properties [13,14]. The presence of the carbamate functional group in these materials allows for the design of innovative polymeric architectures, which differ significantly from amino acid-based polymers, paving the way for novel applications in material science [15].

Given the wide applicability of carbamates, their synthesis has been extensively investigated, with a particular focus on optimizing reaction conditions, improving yields, and minimizing environmental impact. Traditional synthetic routes include the phosgenation of amines, a well-established but hazardous method due to the high toxicity of phosgene [16]. Alternative approaches have emerged, including oxidative carbonylation and the *N*-acylation of amino alcohols, both of which provide more sustainable and selective pathways for carbamate formation [17]. One notable advancement involves the use of CO_2_ as a carbonyl source in combination with propargyl alcohols and amines. This method, catalyzed by a Cu_2_O/biobased ionic liquid system, achieves 100% atom economy while avoiding hazardous carbon sources such as CO and phosgene [18]. Similarly, a three-component coupling reaction between CO_2_, amines, and alkyl halides has been explored, with polymer-supported DBU catalysts facilitating the reaction to achieve high yields while eliminating classical purification steps [19]. Additionally, a solvent-free synthesis using sodium cyanate, phenol/alcohol, and trichloroacetic acid (TCA) has demonstrated efficiency in producing primary carbamates in high purity [20]. In medicinal chemistry, carbamates have proven to be crucial in drug development due to their structural stability and ability to interact with biological targets. The synthesis of carbamate derivatives from 4-amino-1,2,4-triazoles using phenyl chloroformate in acetonitrile with potassium carbonate is one such example. This reaction produces both mono- and bicarbamate derivatives, optimizing the reaction conditions to achieve high yields [21]. Despite the advancements in synthetic methods, challenges remain in optimizing reaction conditions and improving the sustainability of these processes. The ongoing development of greener and more efficient methodologies continues to be a focal point in the field, driven by the increasing demand for carbamate-based compounds in various industries.

Among the methods of synthesizing carbamates, homogeneous catalysis using transition metal complexes has demonstrated remarkable efficiency and selectivity in promoting carbamate synthesis under relatively mild conditions [22]. One specific reaction that has garnered attention is the synthesis of (*R*)-methyl-(2-hydroxy-1-phenylethyl)carbamate from (*R*)-(-)-2-phenylglycinol and methyl chloroformate. This reaction, catalyzed by calcium hydroxide, results in the formation of a methyl carbamate derivative that exhibits supramolecular interactions through hydrogen bonding [23]. The stability of this structure makes it a promising candidate for applications in drug design, where carbamates have been shown to enhance pharmacokinetic and pharmacodynamic profiles [24,25].

Palladium-catalyzed reactions are particularly attractive due to their ability to facilitate key bond-forming steps, such as oxidative addition, ligand exchange, and reductive elimination, all of which are crucial for C–N and C–O bond formation [26]. The versatility of palladium complexes allows for modifications in ligand environments, enabling fine-tuned reactivity and improved selectivity in catalytic processes. In this context, Tetrakis(triphenylphosphine)palladium (Pd(PPh_3_)_4_) has been widely employed as a homogeneous catalyst for various organic transformations, including the synthesis of carbamates. Its ability to mediate cross-coupling reactions under mild conditions makes it a valuable tool in modern synthetic chemistry [27].

Despite significant progress in the synthesis of carbamates using heterogenous catalysis, the mechanistic details of these reactions remain poorly understood. While experimental studies have provided insights into reaction intermediates and final products, the precise pathways leading to carbamate formation are often inferred rather than directly observed. This limitation highlights the need for computational studies to complement experimental findings and provide a deeper mechanistic understanding of the reaction.

Computational chemistry, particularly density functional theory (DFT), has emerged as a powerful tool for elucidating reaction pathways at the molecular level. DFT calculations enable the identification of key intermediates, the determination of transition states, and the quantification of activation energies, all of which contribute to a more comprehensive understanding of reaction pathways [28]. By modeling potential energy surfaces and electronic structures, computational studies can provide critical insights that are often inaccessible through experimental techniques alone.

In this study, we investigate the formation of (*R*)-methyl-(2-hydroxy-1-phenylethyl)carbamate through homogeneous catalysis using Pd(PPh_3_)_4_ as a catalyst in tetrahydrofuran (THF) as the reaction medium. The importance of studying catalysts for the synthesis of (*R*)-methyl-(2-hydroxy-1-phenylethyl)carbamate cannot be overstated. Catalysts, particularly transition metal complexes like Pd(PPh_3_)_4_, play a fundamental role in overcoming the energetic barriers associated with key reaction steps such as dehydrogenation and chlorine elimination. The use of catalysts is essential for improving reaction efficiency, lowering activation energies, and ensuring the selective formation of the desired product. Additionally, understanding the catalytic pathway provides a foundation for designing more effective catalysts that could enhance yield, reduce reaction time, and minimize environmental impact. This knowledge is particularly valuable for the development of more sustainable synthetic methodologies applicable to various organic transformations.

Previous experimental work has provided evidence for the formation of the carbamate product through infrared spectroscopy (IR) and nuclear magnetic resonance (NMR) analyses, confirming the presence of various reaction intermediates [29]. However, the pathway underlying the formation of the carbamate has yet to be fully elucidated. To address this knowledge gap, we perform a computational study aimed at evaluating the most probable mechanistic routes leading to carbamate formation. The primary objectives of this computational study are to identify key reaction intermediates, determine the relative stability of different mechanistic pathways, and evaluate the thermodynamic and kinetic feasibility of each step in the reaction.

This research contributes to the broader field of catalytic carbamate synthesis by offering mechanistic insights that could aid in the optimization of synthetic methodologies. The findings of this study are expected to have implications for the rational design of catalytic systems, particularly in the development of more efficient and sustainable strategies for carbamate production. Understanding the reaction pathway at a fundamental level could lead to improvements in catalyst performance, reaction efficiency, and selectivity, ultimately enhancing the applicability of palladium-catalyzed carbamate synthesis in industrial and pharmaceutical settings.

## 2. Results

### 2.1. Identified Species Experimentally and Computationally

Several intermediate species have been identified by NMR in the synthesis of (*R*)-methyl-(2-hydroxy-1-phenylethyl)carbamate (compound (**3**)) from (*R*)-(-)-2-phenylglycinol (compound (**1**)) and methyl chloroformate (compound (**2**)). Based on these findings, possible reaction pathways considering these species were proposed. Molecular modeling confirmed the existence of both the reactants and the product molecules. Additionally, several intermediate species were proposed to describe the complete synthesis pathway. To further support the possible existence of these intermediates, vibrational frequency calculations were performed. The results indicated that all frequencies were positive, confirming that these species correspond to energy minima.

#### 2.1.1. Reactant and Product Molecules

As a reactant in the reaction, (*R*)-(-)-2-phenylglycinol was theoretically characterized using IR and NMR techniques. Table 1 presents the computational data of this molecule, compared with the corresponding experimental values (Figures S1 and S2 and Tables S1 and S2).

**Figure 1:**
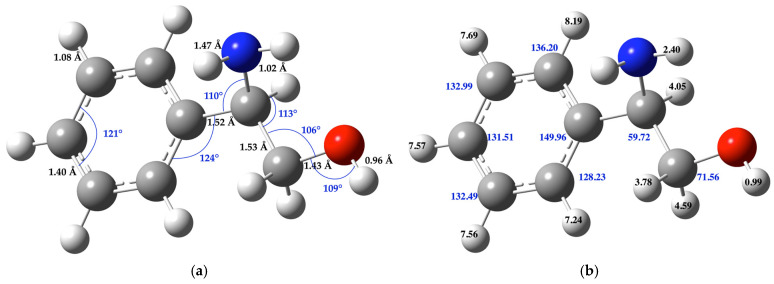
(**a**) **Molecular structure:** Bond distances (black numbers) and angles (blue numbers) (**b**) **NMR information:** Carbon (blue numbers) and hydrogen atoms (black numbers) of the (*R*)-(-)-2-phenylglycinol molecule considered for the chemical shift calculation. The gray balls represent carbon atoms, the white balls are hydrogen atoms, the red balls are oxygen atoms, and the blue balls are nitrogen atoms. All aryl C–C bond distance is 1.40 Å and the C–H bond distance in the ring is 1.08 Å (Table S3 presents the Cartesians coordinates of this compound).

Figure 1 illustrates the molecular structure of (*R*)-(-)-2-phenylglycinol, the calculated chemical shift value of each carbon (C) and hydrogen (H) atoms is indicated at the respective atom. The bond distances and angles of the molecule are expressed in the figure.

Likewise, as a reactant in the reaction, methyl chloroformate was theoretically characterized using IR and NMR techniques. Table 2 presents the computational data for this molecule, compared with the corresponding experimental values (Figures S3 and S4).

**Figure 2:**
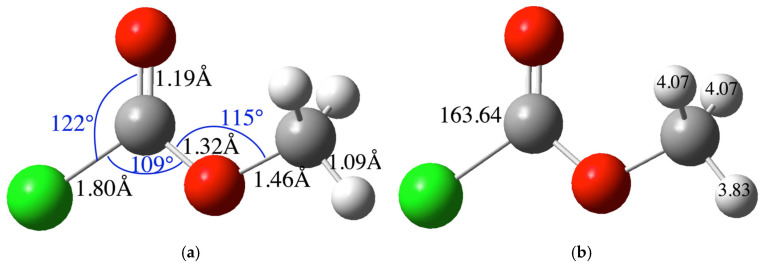
(**a**) **Molecular structure:** Bond distances (black numbers) and angles (blue numbers); (**b**) **NMR information:** Carbon and hydrogen atoms of the chloroformate molecule considered for the chemical shift calculation. The gray balls represent carbon atoms, the white balls are hydrogen atoms, the red balls are oxygen atoms, and green balls are chlorine atoms.

Figure 2 illustrates the molecular structure of methyl chloroformate, the calculated chemical shift value of each carbon (C) and hydrogen (H) atoms is indicated at the respective atom. The bond distances and angles of the molecule are expressed in the figure (Table S4 shows the Cartesian coordinates of this compound).

On the other hand, as the reaction product, (*R*)-methyl-(2-hydroxy-1-phenylethyl)carbamate was theoretically characterized using IR and NMR techniques. Table 3 provides the computational data for this molecule, compared with experimental results (Figures S5 and S6 and Tables S3 and S4).

**Figure 3:**
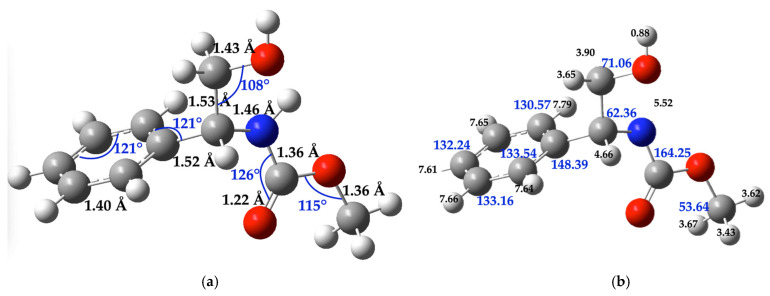
(**a**) **Molecular structure:** Bond distances (black numbers) and angles (blue numbers); (**b**) **NMR information:** Carbon (blue numbers) and hydrogen atoms (black numbers) of the (*R*)-(-)-2-phenylglycinol molecule considered for the chemical shift calculation. The gray balls represent carbon atoms, the white balls are hydrogen atoms, the red balls are oxygen atoms, and the blue balls are nitrogen atoms. All aryl C–C bond distance is 1.40 Å and the C–H bond distance in the ring is 1.08 Å.

Figure 3 displays the molecular structure of (*R*)-methyl-(2-hydroxy-1-phenylethyl)carbamate, the calculated chemical shift value of each carbon (C) and hydrogen (H) atoms is indicated at the respective atom. The bond distances and angles of the molecule are expressed in the figure (Table S7 shows the Cartesian coordinates of this compound).

The concordance between the experimental and computational values of the chemical shift and IR vibrational frequencies indicates the comparability of both types of data, additionally corroborating that the employed chemical models are adequate.

Finally, Table 4 presents the experimental and calculated structural data for (*R*)-methyl-(2-hydroxy-1-phenylethyl)carbamate. The differences between calculated and experimental values are unsignificant, around 0.03 Å in bond distances and 3° in angles.

#### 2.1.2. Tetratikis(trifenilfosfina)palladium Catalyst

The calculations indicated that (*R*)-(-)-2-phenylglycinol and methyl chloroformate do not spontaneously form the carbamate, as the interaction energy between both species is −3.7 kcal/mol. Therefore, the use of a catalyst is necessary. Experimental assays identified Tetratikis(trifenilfosfina)palladium (Pd(PPh_3_)_4_) as the most effective homogeneous catalyst. It is important to clarify that the term ‘interaction energy’ used in this study refers to the energy associated with the stabilization of the complex formed between the interacting species, and should not be confused with the energy barrier or activation energy, which represents the minimum energy required for the reaction to proceed.

However, the interaction of compound (**1**) and compound (**2**) with the catalyst does not spontaneously lead to the formation of the carbamate (compound (**3**)), as the interaction energy is −10.4 kcal/mol, only slightly higher than that of the uncatalyzed system. The calculated energy of Pd(PPh_3_)_4_ is −2.68 × 10^−6^ kcal/mol and its optimized structure consist of three PPh_3_ ligands surrounding the Pd atom (Pd–P distance = 2.39 Å), while the fourth PPh_3_ ligand is more distance (Pd–P distance = 7.49 Å). This structure allows the reactants to approach the catalyst for dehydrogenation or hydrogenation, whereas if all four PPh_3_ ligands were positioned at similar distances around the Pd atom, steric hindrance would likely prevent interaction between the reactants and the catalytic center.

#### 2.1.3. Intermediates

The proposed reaction pathway involves several intermediates, including 4-phenyl-oxazolidine (compound **6**). To confirm that this intermediate corresponds to an energy minimum, vibrational frequency calculations were performed. In all cases, the vibrational frequencies were positive, indicating that the intermediate is stable energy minima. Table 5 shows the experimental and calculated structural data of the 4-phenyl-oxazolidine intermediate, compared with experimental results (Figures S7 and S8 and Tables S5 and S6).

### 2.2. Reaction Pathways

#### 2.2.1. Pathway 1

This proposed pathway results in the formation of the carbamate along with an intermediate species and dehydrogenated (*R*)-(-)-2-phenylglycinol. The latter is a particularly interesting reactant as it promotes the cyclic formation of the carbamate through the following steps.

##### Dehydrogenation of (*R*)-(-)-2-Phenylglycinol

**Figure 4:**
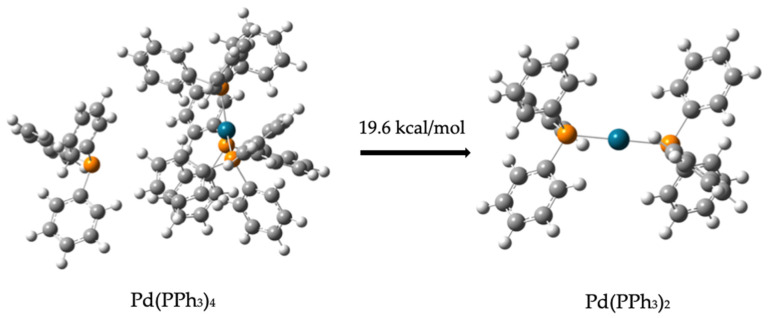
Separation of two phosphine (PPh_3_) ligands from Tetratikis(trifenilfosfina) palladium (Pd(PPh_3_)_4_). The grey balls represent carbon atoms, the white balls are hydrogen atoms, the orange balls are phosphorus atoms, and the turquoise balls are palladium atoms.

Because the reactants do not spontaneously form the carbamate, the first step involves the catalyzed dehydrogenation of (*R*)-(-)-2-phenylglycinol. Prior to this step, the removal of two PPh_3_ ligands from the Pd(PPh_3_)_4_ (compound (**4**)) is necessary to activate the catalyst. In order to preserve the tetravalency of the palladium center, two phosphines (PPh_3_) ligands must be removed, requiring 0.5 kcal/mol and 19.1 kcal/mol, respectively. The total energy required to activate the catalyst for dehydrogenation is 19.6 kcal/mol. Figure 4 demonstrates this process:

Pd(PPh_3_)_4_ → Pd(PPh_3_)_3_ + PPh_3_    0.5 kcal/mol (1)

  Pd(PPh_3_)_3_ → Pd(PPh_3_)_2_ + PPh_3_    19.1 kcal/mol (2)

**Figure 5:**
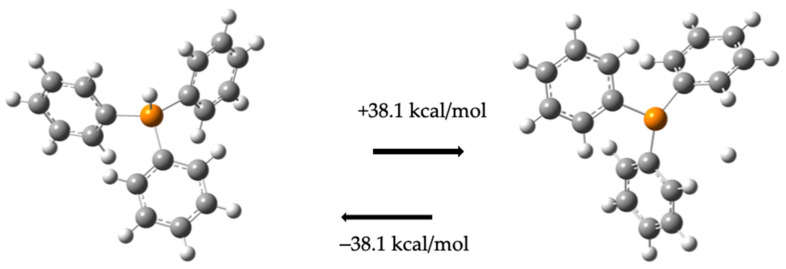
Phosphine molecule hydrogenated and dehydrogenated and the necessary energy for each change. The grey balls represent carbon atoms, the white balls are hydrogen atoms, and the orange balls are phosphorus atoms.

The dissociated phosphine ligand (PPh_3_) interacts with hydrogen atoms (d_P−H_ = 1.42 Å), requiring −38.1 kcal/mol to hydrogenate a phosphine molecule. Thus, separating a hydrogen from (PPh_3_) requires 38.1 kcal/mol, forming the compound (**5**). Figure 5 illustrates both processes. 

**Figure 6:**
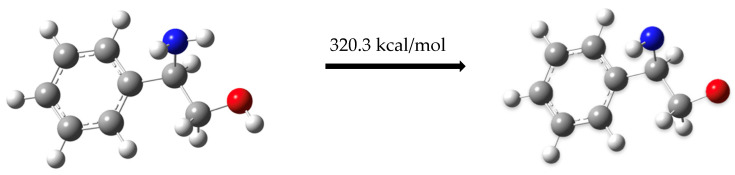
Dehydrogenation of the (*R*)-(-)-2-phenylglycinol molecule.

Next, the palladium center facilitates the removal of two hydrogen atoms from compound (**1**), with each hydrogen binding to a phosphine molecule, yielding an energy of –76.2 kcal/mol. Figure 6 presents the dehydrogenation process of compound (**1**) and the associated energy requirements:

C_6_H_5_CH(NH_2_)CH_2_OH → C_6_H_5_CH(NH)CH_2_OH   158.0 kcal/mol (3)

C_6_H_5_CH(NH)CH_2_OH → C_6_H_5_CH(NH)CH_2_O     162.3 kcal/mol (4)

2 (PPh_3_ → HPPh_3_)                2(−38.1 kcal/mol) (5)

The total energy involved in these steps is 320.3 kcal/mol − 76.2 kcal/mol = 244.1 kcal/mol.

##### Formation of Compounds (**7**) and (**8**) as Intermediate Species

**Figure 7:**
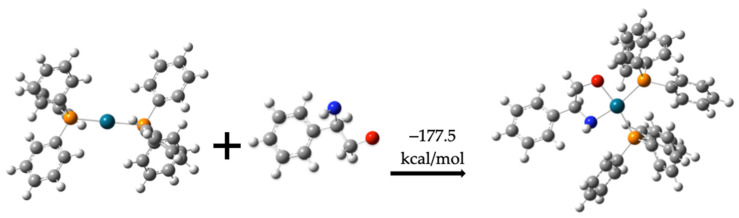
Formation of molecule (**7**) from dehydrogenated molecule (**1**) and Pd(PPh_3_)_2_.

The dehydrogenated compound (**1**) binds to Pd(PPh_2_), forming compound (**7**), releasing −177.5 kcal/mol. Figure 7 illustrates the formation of this intermediate.

**Figure 8:**
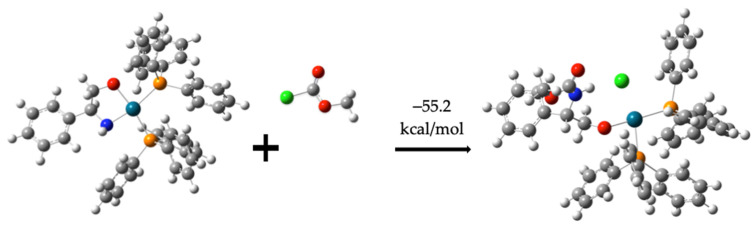
Formation of the molecule (**8**).

Subsequently, compound (**7**) interacts with compound (**2**), leading to the formation of intermediate (**8**). This step is spontaneous, with an interaction energy of −55.2 kcal/mol, as shown in Figure 8. During this process, the chlorine atom form compound (**2**) is eliminated.

At this stage, compound (**8**) can proceed via two possible reaction routes: (a) the formation of compound (**3**), the carbamate, or (b) the formation of intermediate species (**10**), which is experimentally identified and plays a role in regenerating compound (**1**).

C_6_H_5_CH(NH)CH_2_O-Pd-(PPh_3_)_2_ + C_2_H_3_ClO_2_ *→*                       
C_6_H_5_CH(NH)CH_2_O-Pd-(PPh_3_)_2_-C_2_H_3_ClO_2_   −55.2 kcal/mol (6)

##### Formation of Compound (**3**)

**Figure 9:**
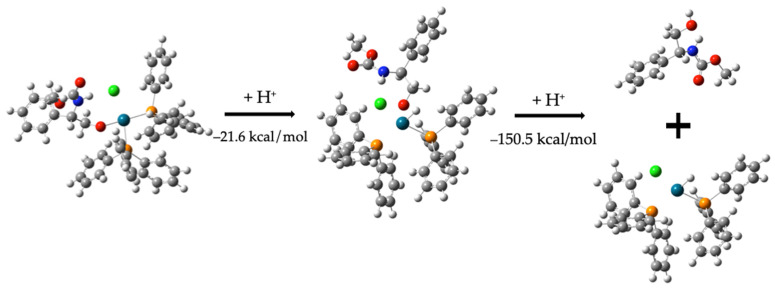
Hydrogenation of compound (**9**) to form compound (**3**), the carbamate.

The final step involves hydrogenation at the -NH and -O centers, leading to carbamate formation and the concurrent dehydrogenation of two HPPh_3_ molecules. The conversion of compound (**8**) to the final carbamate requires a cation (H^+^) to complete the valence of Pd. Since the interaction between Pd and free H atom is more favorable than between Pd and O bonded to the molecule, the Pd–O bond is broken, releasing the molecule and forming the dehydrogenated carbamate. Consequently, an additional H^+^ cation is required. These two protons are supplied from two H-PPh_3_ species, requiring 76.2 kcal/mol (Figure 9).

C_6_H_5_CH(NH)CH_2_O-Pd-(PPh_3_)_2_-C_2_H_3_ClO_2_ + 2H+ *→*                   H-Pd-(PPh_3_)_2_Cl + C_6_H_5_C_2_H_3_OH(NH)C_2_H_3_O_2_   −95.9 kcal/mol (7)

The total energy involved in these steps is 76.2 kcal/mol − 21.6 kcal/mol − 150.5 kcal/mol = –95.9 kcal/mol.

Considering all steps, including the dehydrogenation of compound (**1**) and catalyst modifications, the overall energy of Pathway 1 is −64.9 kcal/mol.

##### Alternative Pathway: Formation of Compound (**10**)

Another possible pathway involves the formation of compound (**10**), an intermediate that can subsequently react with compound (**7**), leading to the formation of other intermediates. This cycle contributes to the production of compound (**7**), an additional identified intermediate compound (**6**), and the dehydrogenated compound (**1**), effectively regenerating reactants and sustaining the catalytic cycle.

#### 2.2.2. Pathway 2

The presence of compound (**6**) (4-phenyl-oxazolidine) in the reaction (see Section 2.1), necessitated the proposal of an alternative and complementary reaction pathway to Pathway 1. Moreover, the formation of additional intermediates must lead to the regeneration of one of the reactants, compound (**1**), ensuring the sustainability of the catalytic cycle.

##### Formation of Compound (**10**) from Compound (**8**)

**Figure 10:**
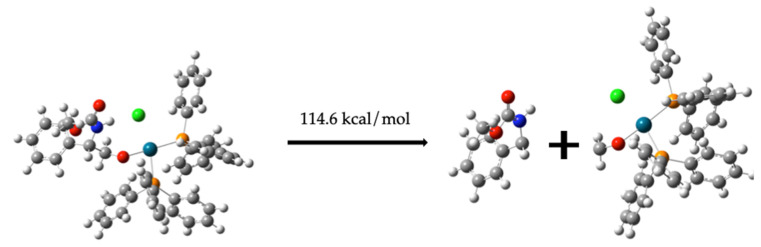
Separation of compound (**10**) from compound (**8**).

Compound (**8**) is expected to form compound (**10**), as illustrated in Figure 10, requiring an energy of 114.6 kcal/mol for the transformation.

C_6_H_5_CH(NH)CH_2_O-Pd-(PPh_3_)_2_-C_2_H_3_ClO_2_ *→*                       C_6_H_5_CH(NH)CH_2_OC = O + Pd-(PPh_3_)_2_-OCH_2_Cl   114.6 kcal/mol (8)

##### Formation of Compound (**11**) from Compounds (**7**) and (**10**)

**Figure 11:**
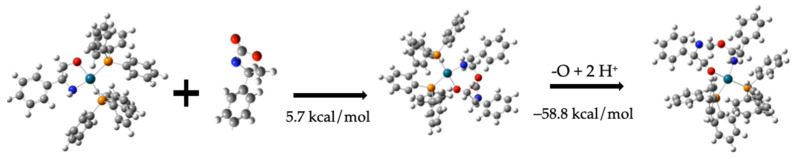
Formation of compound (**12**) from compound (**11**) and compound (**10**).

Compound (**11**) formation from compounds (**7**) and (**10**) requires 5.7 kcal/mol. Compound (**11**) is proposed as a precursor for compound (**6**) (4-phenyl-oxazolidine), a product identified by RMN analysis. To achieve this transformation, an oxygen atom must be removed from compound (**11**), followed by hydrogenation or the corresponding carbon. These two steps are illustrated in Figure 11.

The oxygen removal step from compound (**11**) requires 204.8 kcal/mol, while the subsequent hydrogenation step releases −263.8 kcal/mol, resulting in a net energy of −58.8 kcal/mol. Thus, the total energy required to convert compound (**11**) into compound (**12**) is −53.1 kcal/mol, including the combination of compounds (**7**) and (**10)**.

C_6_H_5_CH(NH)CH_2_O-Pd-(PPh_3_)_2_ + C_6_H_5_CH(NH)CH_2_OC=O →

C_6_H_5_CH(NH)CH_2_O-Pd-(PPh_3_)_2_C_6_H_5_CH(NH)CH_2_OC=O   5.7 kcal/mol (9)

C_6_H_5_CH(NH)CH_2_O-Pd-(PPh_3_)_2_C_6_H_5_CH(NH)CH_2_OC=O–O + 2H^+^ →

C_6_H_5_CH(NH)CH_2_O-Pd-(PPh_3_)_2_C_6_H_5_CH(NH)CH_2_OCH_2_   −58.8 kcal/mol (10)

##### Regeneration of Dehydrogenated Compound (**1**) and Formation of Compound (**6**)

**Figure 12:**
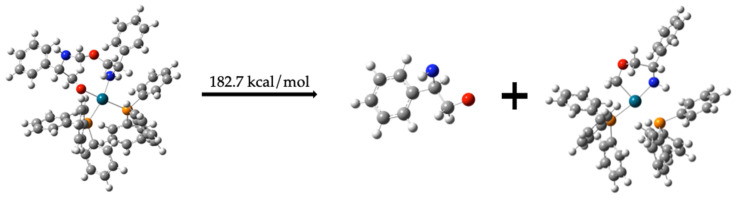
Formation of dehydrogenated compound (**1**) from compound (**12**).

Compound (**12**) is a key intermediate because it can generate the dehydrogenated compound (**1**), which subsequently reacts with Pd-(PPh_3_)_2_ to form compound (**7**). This transformation requires 182.7 kcal/mol, which is significantly lower than the energy required for the catalyzed dehydrogenation of compound (**1**) by Pd-(PPh_3_)_2_ (see Figure 12).

C_6_H_5_CH(NH)CH_2_O-Pd-(PPh_3_)_2_C_6_H_5_CH(NH)CH_2_OCH_2_ *→*

C_6_H_5_CH(NH)CH_2_O + C_6_H_5_CH(NH)CH_2_OCH_2_-Pd-(PPh_3_)_2_   182.7 kcal/mol (11)

**Figure 13:**
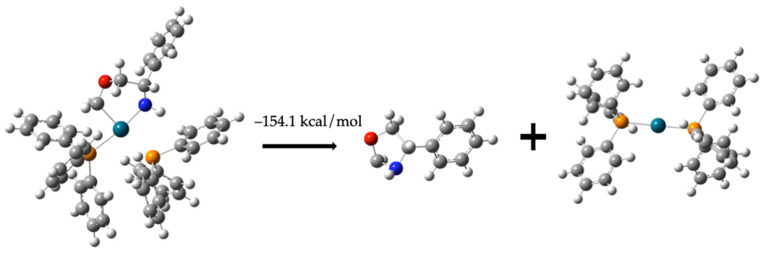
Formation of compound (**6**).

Finally, the intermediate can transform into compound (**6**) with an energy value of −62.4 kcal/mol regenerating the catalyst Pd-(PPh_3_)_2_. The formation of the cyclic compound (**6**) requires an additional −91.7 kcal/mol (see Figure 13).

C_6_H_5_CH(NH)CH_2_OCH_2_-Pd-(PPh_3_)_2_ *→*                        C_6_H_5_CH(NH)CH_2_OCH_2_ + Pd-(PPh_3_)_2_   −154.1 kcal/mol (12)

**Figure 14:**
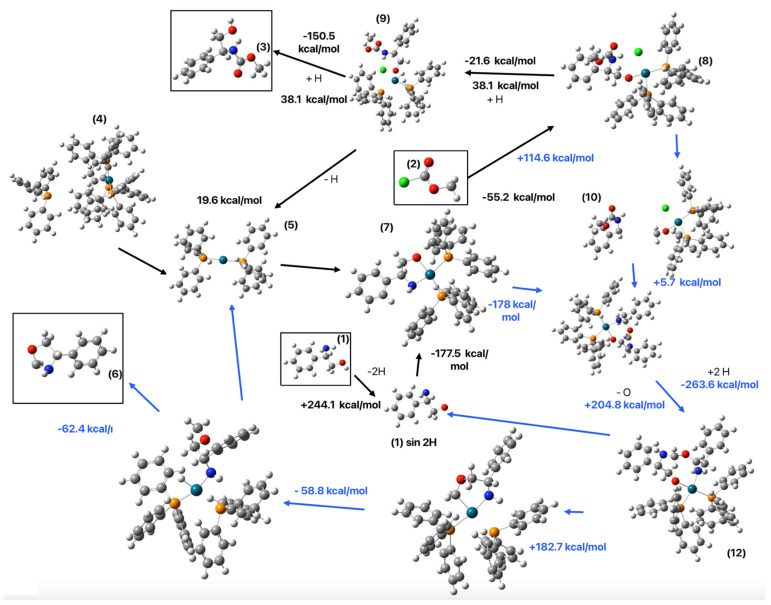
Combination of Pathways 1 and 2 to form compound (**3**). The identified molecules by NMR are indicated with a square. Each step of Pathway 1 relates with a black arrow and the steps of Pathway 2 relate with blue arrows. The gray balls represent carbon atoms, the white balls are hydrogen atoms, the red balls are oxygen atoms, the blue balls are nitrogen atoms, the orange balls are phosphorus atoms, and the turquoise balls are palladium atoms.

Taking into account all the steps of Pathway 2, the necessary energy is 90.1 kcal/mol, but despite the fact that this set of reaction is endothermic and not spontaneous, this pathway avoids the need for the direct dehydrogenation of compound (**1**), saving 244.1 kcal/mol, which would otherwise be required in Pathway 1. As a result, the overall energy of Pathway 1 (−64.9 kcal/mol) and Pathway 2 (90.1 kcal/mol) together lead to a net reaction energy of −218.9 kcal/mol. Figure 14 shows the two pathways and the energy of each step.

## 3. Discussion

The computational findings presented in this study provide a detailed understanding of the formation pathway of (*R*)-methyl-(2-hydroxy-1-phenylethyl)carbamate and highlight the fundamental role of Pd(PPh_3_)_4_ as a homogeneous catalyst in enabling this conversion. The results confirm that the direct reaction between (*R*)-(-)-2-phenylglycinol (compound **1**) and methyl chloroformate (compound **2**) is insufficient to promote carbamate formation, as evidenced by the weak interaction energy between the reactants (−3.7 kcal/mol). This weak interaction suggests that the reactants alone cannot proceed effectively toward carbamate formation without additional catalytic assistance, especially since the energy to break the necessary bonds in both molecules and form the new species is 150.4 kcal/mol. It is important to clarify that the low interaction energy refers to the initial encounter between the reactants and does not necessarily imply the thermodynamic unfavourability of the overall process. Instead, it highlights the need for a catalyst to lower the activation energy (ΔG‡), thereby enhancing the reaction rate and enabling the formation of the desired carbamate (compound **3**). Therefore, through computational modeling, a multi-step reaction pathway was proposed, demonstrating that ligand dissociation, intermediate formation, and final hydrogenation play essential roles in the catalytic efficiency of the conversion. The results further confirm that Pd(PPh_3_)_4_ provides a suitable environment for the reaction, stabilizing intermediate species and enabling the sequential conversions required for carbamate synthesis.

One of the most important aspects of this study is the role of Pd(PPh_3_)_4_ in facilitating the reaction. Unlike conventional catalysts that promote single-step transformations, Pd(PPh_3_)_4_ orchestrates a sequence of elementary steps that collectively lead to carbamate formation. Thus, a key observation is the ligand dissociation process, which significantly influences catalytic efficiency. Computational results indicate that the displacement of a phosphine ligand creates an open coordination site on the palladium center, allowing reactants to approach and interact with the catalyst. This open coordination site is essential for enabling dehydrogenation and chlorine elimination, both of which are critical transformations required to generate the carbamate product. Additionally, the interaction between the palladium center and the reaction intermediates suggests that Pd(PPh_3_)_4_ acts as both an electron donor-acceptor and a stabilizing agent for highly reactive species. The formation of a palladium complex with the dehydrogenated intermediate of (*R*)-(-)-2-phenylglycinol is highly exergonic (−177.5 kcal/mol), demonstrating that Pd(PPh_3_)_4_ effectively stabilizes transition states and charged intermediates that would otherwise be too unstable to persist in solution. Moreover, the final hydrogenation step, which regenerates the palladium species and produces the carbamate product, is also highly exergonic (−265.7 kcal/mol). This indicates that, once the key transformations occur, the reaction proceeds with thermodynamic favorability, provided that the catalytic cycle is efficiently conserved. These findings provide strong computational evidence that Pd(PPh_3_)_4_ is a highly effective catalyst for this transformation, though alternative catalytic systems with modified ligands could potentially enhance reactivity and selectivity.

In addition to the use of Pd(PPh_3_)_4_, other catalysts have been investigated for similar reactions involving the synthesis of carbamate compounds. For instance, Mendoza Herrera et al. (2024) [23] successfully employed calcium hydroxide (Ca(OH)_2_) as a heterogeneous catalyst in the reaction between (R)-2-phenylglycinol and methyl chloroformate, resulting in the formation of a carbamate derivative. The catalyst demonstrated good yields under mild conditions and promoted the formation of supramolecular chains through hydrogen bonding interactions. This alternative catalytic approach highlights the potential of employing inexpensive and environmentally friendly catalysts in such reactions. Although Pd(PPh_3_)_4_ was selected for this study due to its effectiveness in promoting dehydrogenation and dichlorination processes, the possibility of exploring alternative catalysts remains open. Future studies could focus on evaluating other transition metal complexes, metal oxides, or even organic catalysts that might offer improved efficiency, selectivity, or sustainability. The computational approach used in this work provides a suitable platform for screening such alternatives before experimental validation.

On the other hand, the energy analysis of the two proposed pathways offers significant insights into the feasibility of carbamate formation under the investigated catalytic conditions. Pathway 1, which involves the direct dehydrogenation of (*R*)-(-)-2-phenylglycinol, results in a net energy change of −84.7 kcal/mol, indicating a thermodynamically favorable route. In contrast, Pathway 2 follows an alternative sequence that includes the formation of additional intermediates, leading to a total energy of 90.1 kcal/mol. Although the positive energy value of Pathway 2 may initially suggest that it is not a favorable route, its contribution to regenerating key intermediates makes it a fundamental component of the overall catalytic cycle. Indeed, Pathway 2 contributes significantly to the overall catalytic cycle because it regenerates dehydrogenated compound (**1**) and intermediate compound (**7**) while producing compound (**10**). A fundamental implication of this reaction sequence is that Pathway 2 prevents the need for the direct dehydrogenation of compound (**1**), saving 244.1 kcal/mol, which would otherwise be required in Pathway 1. As a result, the overall energy of Pathway 1 (−84.7 kcal/mol) and Pathway 2 (90.1 kcal/mol) together lead to a net reaction energy of −238.7 kcal/mol. This computational validation confirms that the reaction can proceed through both Pathway 1 and Pathway 2, efficiently leading to carbamate formation while ensuring catalyst regeneration. The balance between the two pathways provides a deeper understanding of how transition metal catalysis enables complex multi-step transformations, with energy compensation across different mechanistic routes leading to the thermodynamic favorability of the overall process.

Regarding intermolecular interactions and product stability, it is evident that the stability of the final carbamate product is strongly influenced by C–N bond formation, a key step that determines the viability of the reaction. Computational results confirm that the C-N bond is fundamental to product stability and directly affects the overall reaction pathway. These results align with previous studies, particularly those of Herrera et al. [23], where similar C–N interactions were analyzed in related catalytic systems. The computational data indicate that electronic factors around the nitrogen atom play a crucial role in modulating the C–N bond strength. The partial charge distribution on nitrogen, in combination with metal-catalyzed dehydrogenation, facilitates efficient carbamate formation, confirming that palladium catalysis enhances electronic density and promotes amine reactivity.

Finally, the mechanistic insights from this study offer a basis for optimizing catalytic systems for carbamate synthesis, emphasizing the critical role of ligand dissociation in catalytic activity. Future research should explore ligand modifications, such as bulkier phosphine ligands to regulate dissociation rates and improve the reactivity and selectivity, or electronic modifications using electron-withdrawing or electron-donating groups to alter reaction energetics. Beyond ligand modifications, alternative catalytic systems, including bimetallic or heterogeneous palladium catalysts, could improve turnover frequencies and stability, while solid-supported palladium catalysts may improve recyclability and cost-effectiveness for large-scale applications. Furthermore, further computational and experimental investigations are essential to refine the reaction pathways and identify new intermediates, with advanced spectroscopic techniques such as in situ IR, NMR and Raman spectroscopy providing deeper insights into the transition states.

## 4. Materials and Methods

In this study, the Gaussian 16 [30] Revision A.03 software package (Gaussian, Inc., Wallingford, CT, USA) was used. Based on the experimental data, all chemical species were modeled using GaussView 6 [31]. The root-mean-square convergence criterion for the density matrix in the self-consistent field (SCF) iteration was set to 10^−14^ a.u., targeting an energy convergence threshold of at least 10^−15^ a.u. (GAUSSIAN keyword: SCF = tight). The default convergence criteria recommended by Gaussian16 were employed to improve the reliability and accuracy of the calculations [1]. These stringent parameters ensure that the optimized structures correspond to genuine energy minima, which is critical for accurately characterizing the stability and reaction pathways of molecules.

The structures of all compounds were full optimized from the experimental data, using the Becke’s three-parameter (B3) hybrid exchange functional with the correlation functional of Lee Yang and Parr (LYP), within density functional theory (DFT) [32]. Grimme’s empirical dispersion correction (D3) was applied to the B3LYP functional to accurately account for dispersion interactions. The B3LYP functional was chosen for this study due to its well-documented reliability in providing a balanced description of electronic, geometric, and energetic properties of organic systems and transition metal complexes. Numerous studies have demonstrated its effectiveness in accurately predicting structural parameters and reaction energetics, particularly when combined with appropriate basis sets [33,34,35]. The chemical structures were optimized using Berny’s algorithm, and the multiplicity for each state was set to the lowest, generally in the singlet state. The 6-311G* basis set was employed for main-group elements (C, P, O, N, H and Cl), as it offers a good compromise between computational cost and accuracy, allowing for the precise calculation of geometries and vibrational frequencies [36]. For the palladium atom, the Stuttgart RSC 1997 (sdd) pseudopotential was selected to efficiently account for relativistic effects, which is essential when dealing with heavy transition metals like palladium [37]. While it is acknowledged that higher-level methods, such as post-Hartree–Fock approaches or advanced hybrid functionals with larger basis sets, could potentially enhance accuracy, their application is often limited by a significantly increased computational cost. Given the complexity of the studied systems and the extensive exploration of potential reaction pathways, the B3LYP/6-311G* combination provides reliable results within a reasonable computational effort. This approach has been successfully applied to similar systems involving transition metal catalysis, supporting its suitability for the present study [38,39]. Although the B3LYP functional has been successfully employed to describe the catalytic pathway of Pd(PPh_3_)_4_, it is recognized that more modern functionals, such as M06-2X and wB97M-V, could offer greater accuracy, particularly in capturing dispersion interactions and non-local correlation effects. These functionals are specifically designed to provide improved the descriptions of reaction energetics and long-range interactions. However, their higher computational cost makes them less practical for extensive mechanistic studies. Future investigations could benefit from employing these advanced functionals for benchmarking and comparative analysis.

Finally, the NMR chemical shifts reported were calculated using the GIAO-B3LYP method [40], and all chemical shift values were corrected with Tetramethylsilane (TMS). The structural optimization and computational IR spectra were calculated considering Tetrahydrofuran (THF) as solvent, in similar way to the experimental conditions, and chloroform was considered as solvent in NMR chemical shifts calculations.

## 5. Conclusions

This study presents a comprehensive computational analysis of the Pd(PPh_3_)_4_-catalyzed formation of (R)-methyl-(2-hydroxy-1-phenylethyl)carbamate, providing mechanistic insights into the reaction pathway, catalyst function, and energy profile. The findings confirm that Pd(PPh_3_)_4_ plays a crucial role in stabilizing intermediates, reducing activation barriers, and facilitating key transformations such as dehydrogenation and chlorine elimination. The proposed multi-step reaction pathway is energetically favorable, as Pathway 1 and Pathway 2 together result in a net reaction energy of –238.7 kcal/mol. This computational validation supports the feasibility of both pathways, emphasizing their contributions to catalyst regeneration and product formation.

By comparing these findings with previous studies, particularly Herrera et al. [21], this study highlights the significance of C–N bond formation and confirms the role of palladium catalysis in enhancing electronic and steric interactions during carbamate synthesis. These insights pave the way for future catalyst optimization, providing a foundation for developing more efficient, selective, and sustainable catalytic strategies for industrial and pharmaceutical applications.

## Figures and Tables

**Table 1:** Experimental and calculated of H and C chemical shift of (*R*)-(-)-2-phenylglycinol.

Chemical Shift H (ppm)		Chemical Shift C (ppm)		Calculated IR Frequencies (cm^−1^)
Experimental	Calculated	Experimental	Calculated	
2.61	2.4	67.84	71.56	3543–3465 (N-H)
3.54	3.78	57.37	59.72	3802 (O-H)
3.71	4.59	142.46	149.96	1537 (C=C)
4.02	4.05	126.53–128.58	128.23–136.20	1070 (C-C)
7.24–7.28	7.57			888 (C-N)
7.29–7.35	7.24–8.19			1045 (C-O)

**Table 2:** Experimental and calculated of H and C chemical shift of the methyl chloroformate.

H Chemical Shift (ppm)		C Chemical Shift (ppm)		Calculated IR Frequencies (cm^−1^)
Experimental	Calculated	Experimental	Calculated	
3.97	3.83–4.07	58.24	61.85	1152 (C-O)
		151.41	163.64	1844 (C=O)
				815 (O=C-O)
				454 (C-Cl)

**Table 3:** Experimental and calculated chemical shifts for H and C atoms of the (*R*)-methyl-(2-hydroxy-1-phenylethyl)carbamate.

H Chemical Shift (ppm)		C Chemical Shift (ppm)		IR Frequencies (cm^−1^)	
Experimental	Calculated	Experimental	Calculated	Experimental	Calculated
3.67	3.43, 3.62	52.35	53.64	3333	3602 (N-H)
3.87	3.65, 3.90	66.52	71.06	1701	1772 (C=O)
4.83	4.66	57.10	62.36	1541	1540 (-C=C-)
	0.88			1260	1264 (C-O)
5.5	5.52			1028	1054 (C-O)
7.29–7.38	7.61–7.79	128.5–128.8	130.57, 132.24	701	771 (=C-H)
		132.05	133.16, 133.25, 133.54		
		132.15	148.39		
		157.09	164.26		

**Table 4:** Experimental and calculated structural data for (*R*)-methyl-(2-hydroxy-1-phenylethyl)carbamate.

Bond	Experimental Bond Distance (Å)	Calculated Bond Distance (Å)	Angle	Experimental (°)	Calculated (°)
N-C	1.430	1.461	N-C-O	109.7	110.1
N-C	1.336	1.363	N-C-O	126.1	125.8
C=O	1.185	1.215	O-C-O	124.2	124.1
C-O	1.353	1.358	C-O-C	116.1	115.5
O-C	1.414	1.436	C-N-C	122.5	120.7
C-C	1.521	1.531	C-C-O	111.7	108.0
C-O(OH)	1.441	1.426			

**Table 5:** Experimental and calculated structural data of 4-phenyl-oxazolidine.

Chemical Shift H (ppm)		Chemical Shift C (ppm)	
Experimental	Calculated	Experimental	Calculated
4.69	4.18,4.78	127.02	86.37
4.36	4.78	56.29	65.28
3.82	3.17,4.54	62.42	76.4
7.30–7.37	7.34–7.70	133.50	151.75
		129.22–129.50	128.67–133.42

## Data Availability

Data are available throughout the manuscript.

## Supplementary Materials

The following supporting information can be downloaded at: https://www.mdpi.com/article/10.3390/molecules30081781/s1, Figure S1. ^1^H NMR spectrum of (R)-(-)-2-phenylglycinol (500 MHz in CDCl_3_ at room temperature, internal reference TMS); Figure S2. ^13^C NMR spectrum of (R)-(-)-2-phenylglycinol (500 MHz in CDCl_3_ at room temperature, internal reference TMS); Figure S3. ^1^H NMR spectrum of methyl chloroformate (500 MHz in CDCl_3_ at room temperature, internal reference TMS); Figure S4. ^13^C NMR spectrum of methyl chloroformate (500 MHz in CDCl_3_ at room temperature, internal reference TMS); Figure S5. ^1^H NMR spectrum of the crude reaction product 8 (500 MHz in CDCl_3_ at room temperature, internal reference TMS); Figure S6. ^13^C NMR spectrum of (R)-methyl-(2-hydroxy-1-phenylethyl)carbamate (500 MHz in CDCl_3_ at room temperature, internal reference TMS); Figure S7. ^1^H NMR spectrum of 4-phenyl-oxazolidine (500 MHz in CDCl_3_ at room temperature, internal reference TMS); Figure S8. ^13^C NMR spectrum of 4-phenyl-oxazolidine. (500 MHz in CDCl_3_ at room temperature, internal reference TMS); Table S1. Main signals of the ^1^H NMR spectrum of (R)-(-)-2-phenylglycinol; Table S2. Main signals of the ^13^C NMR spectrum of (R)-(-)-2-phenylglycinol; Table S3. Cartesian coordinates in Angstroms of (R)-(-)-2-phenylglycinol (compound (**1**)); Table S4. Cartesian coordinates of methyl chloroformate (compound (**2**)); Table S5. Main signals of the ^1^H NMR spectrum of (R)-methyl-(2-hydroxy-1-phenylethyl)carbamate; Table S6. Main signals of the ^13^C NMR spectrum of (R)-methyl-(2-hydroxy-1-phenylethyl)carbamate; Table S7. Cartesian coordinates of (R)-methyl-(2-hydroxy-1-phenylethyl)carbamate (compound (**3**)); Table S8. Main signals of the ^1^H NMR spectrum of 4-phenyl-oxazolidine; Table S9. Main signals of the ^13^C NMR spectrum of 4-phenyl-oxazolidine; Table S10. Cartesian coordinates of 4-Phenyl-oxazolidine (compound (**6**)); Table S11. Cartesian coordinates of Tetratikis(trifenilfosfina)palladium (Pd(PPh_3_)_4_) (compound (**4**)); Table S12. Cartesian coordinates of the compound (**7**), a central intermediate species for Pathways 1 and 2; Table S13. Cartesian coordinates of the compound (**8**), an intermediate species for Pathway 1; Table S14. Cartesian coordinates of the compound (**12**), an intermediate species for Pathway 2.

## Abbreviations

The following abbreviations are used in this manuscript:

DFT	Density Functional Theory
PCM	Polarizable Continuum Model
SCF	Self-Consistent Field
TMS	Tetramethylsilane
THF	Tetrahydrofuran
NMR	Nuclear Magnetic Resonance
IR	Infrared
