# Supplementary material for: Computational Study on the Pd-Catalyzed Pathway for the Formation of (R)-Methyl-(2-Hydroxy-1-Phenylethyl)Carbamate
Source: Molecules. 2025 Apr 16;30(8):1781. doi: 10.3390/molecules30081781 (PMC12029437; doi:10.3390/molecules30081781)
Supplement: Supplementary file 1 [file molecules-30-01781-s001.zip › molecules-3556734-supplementary.pdf]

molecules

MDPI

Supplementary Materials

Computational Study on the Pd-Catalyzed Mechanism for the Formation of (*R*)-Methyl-(2-Hydroxy-1-Phenylethyl)Carbamate

1. Characterization by ^{1}H and ^{13}C NMR

1.1. (R)-(−)-2-phenylglycinol

Figure S1 and **Table S1** show the ^{1}H NMR spectrum of (R)-(−)-2-phenylglycinol. A broad, low-intensity signal appears at 2.61 ppm, corresponding to the two hydrogens of the amine group. At 3.54 and 3.71 ppm, two doublet of doublets are observed, each integrating to one proton. These signals are attributed to the diastereotopic hydrogens of the $-CH_2$ group (Hb1-Hb2), which are near the chiral carbon (Cc). A doublet of doublets at 4.02 ppm corresponds to the hydrogen of the chiral carbon (Hc), integrating to one proton. In the aromatic region, two multiplet signals are visible between 7.24–7.28 ppm and 7.29–7.35 ppm, attributed to the para hydrogen (Hh) and the ortho-meta hydrogens (Hf, Hg) of the phenyl ring, respectively. These signals integrate for 1 and 4 hydrogens, respectively. It is important to note that the signal corresponding to the hydroxyl group (-OH) is not observed. This absence may be due to hydrogen bonding interactions that affect the electronic density, causing the signal to not appear clearly at the baseline.

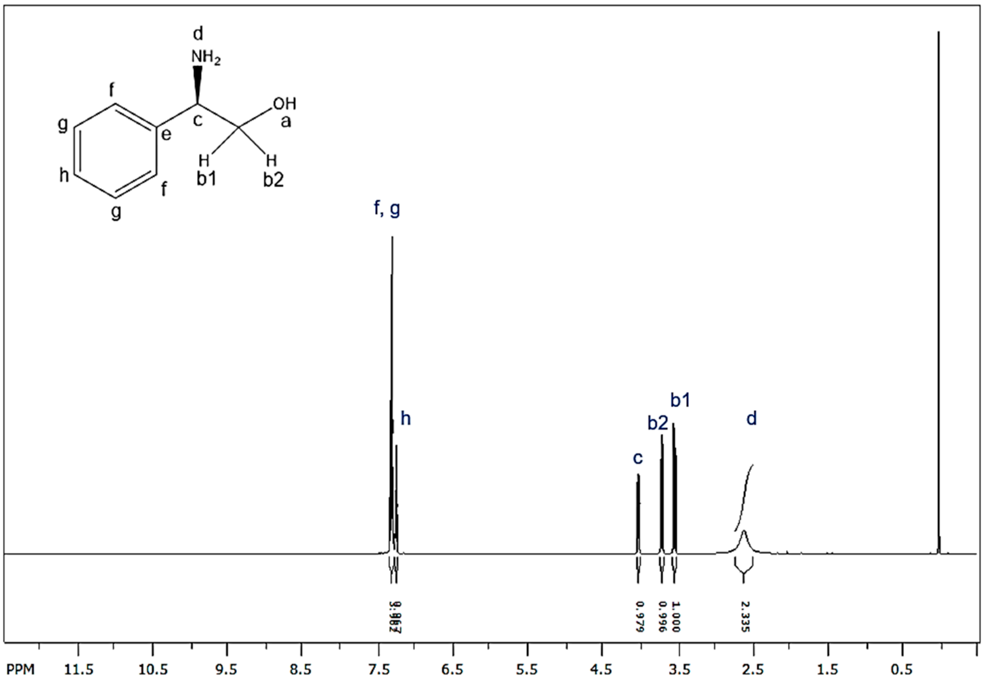

Figure S1. ^{1}H NMR spectrum of (R)-(−)-2-phenylglycinol (500 MHz in $CDCl_3$ at room temperature, internal reference TMS).

Table S1. Main signals of the ^{1}H NMR spectrum of (R)-(−)-2-phenylglycinol.

Signal	Chemical Shift (ppm)	Integral	Multiplicity
Hd	2.61	2	Singlet
Hb1	3.54	1	Doublet of doublets
Hb2	3.71	1	Doublet of doublets
Hc	4.02	1	Doublet of doublets
Hh	7.24-7.28	1	Multiplet
Hf,g	7.29-7.35	4	Multiplet

With respect to the ^{13}C NMR spectrum of (R)-(−)-2-phenylglycinol (see Figure S2), the signal corresponding to the chiral carbon (Cc) is observed at 57.37 ppm. The signal located at 67.84 ppm represents the carbon of the $-CH_2$ group (Cb).

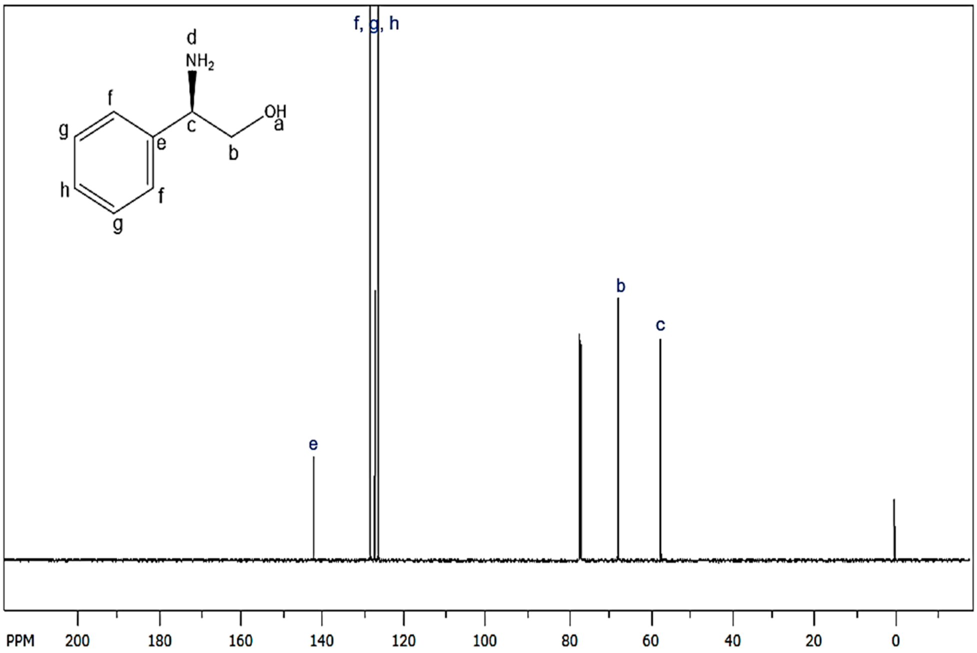

Figure S2. ^{13}C NMR spectrum of (R)-(−)-2-phenylglycinol (500 MHz in $CDCl_3$ at room temperature, internal reference TMS).

The signals observed between 126.53–128.58 ppm correspond to the carbons of the aromatic ring at the ortho, para, and meta positions (Cf, Cg, and Ch). The signal located at 142.46 ppm corresponds to the ipso carbon (Ce). It is important to note that the amine and hydroxyl groups ($-NH_2$ and -OH) do not generate signals in this spectrum since they do not contain carbons. The complete assignment of signals is summarized in **Table S2**.

Table S2. Main signals of the ^{13}C NMR spectrum of (R)-(−)-2-phenylglycinol.

Signal	Chemical Shift (ppm)
Cb	67.84
Cc	57.37
Ce	142.46

Cf,g,h	126.53-128.58

The possition of each atom of the molecule (R)-(-)-2-phenylglycinol (compound (**1**)) in Cartesian coordinates is ilustred in **Table S3**.

Table S3. Cartesian coordinates in Angstroms of (R)-(-)-2-phenylglycinol (compound (**1**)).

Atomic center	x	y	z
O	-2.476662	-2.384607	-0.246929
N	0.023499	-2.030398	1.026737
C	-0.420301	-1.215807	-0.119266
C	0.348634	0.097705	-0.141898
C	-1.940407	-1.061294	-0.187276
C	1.749783	0.048036	-0.162664
C	-0.268187	1.352374	-0.140102
C	2.509608	1.212794	-0.187372
C	0.491634	2.524559	-0.16478
C	1.881312	2.459826	-0.189308
H	-0.138985	-1.781198	-1.017768
H	-2.233431	-0.488917	-1.077027
H	-2.31071	-0.534923	0.703108
H	2.237734	-0.920366	-0.147683
H	-1.348948	1.435892	-0.11955
H	-0.55287	-2.867537	1.052056
H	-0.171172	-1.525089	1.888626
H	3.593153	1.149817	-0.206766
H	-0.009091	3.487638	-0.162986
H	2.471699	3.370268	-0.208842
H	-3.436391	-2.328872	-0.190312

1.2. Methyl Chloroformate

The ^{1}H and ^{13}C NMR spectra of Methyl Chloroformate are shown in Figures S3 and S4, respectively. Due to the simplicity of the molecular structure, very few signals are present in both spectra. In the ^{1}H NMR spectrum (Figure S3), a singlet signal appears at 3.97 ppm, corresponding to the hydrogens of the $-CH_3$ group (Ha), integrating for three protons.

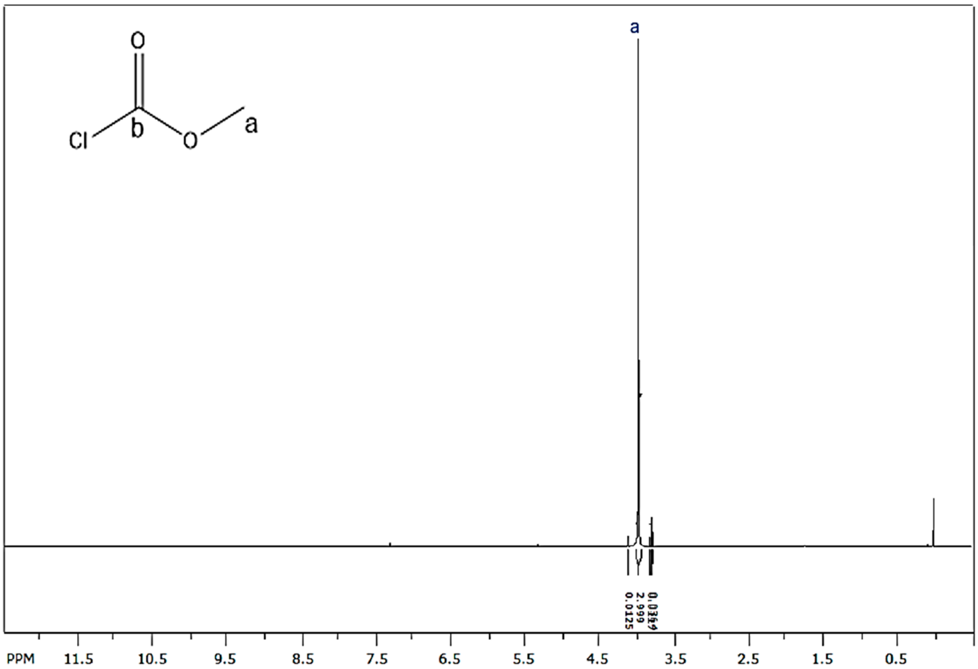

Figure S3. ^{1}H NMR spectrum of Methyl Chloroformate (500 MHz in $CDCl_3$ at room temperature, internal reference TMS).

In the ^{13}C NMR spectrum (Figure S4), two signals are observed: one at 58.24 ppm corresponding to the carbon of the $-CH_3$ group (Ca), and the other at 151.41 ppm corresponding to the carbonyl carbon (Cc). These signals are consistent with previously reported data in the literature.

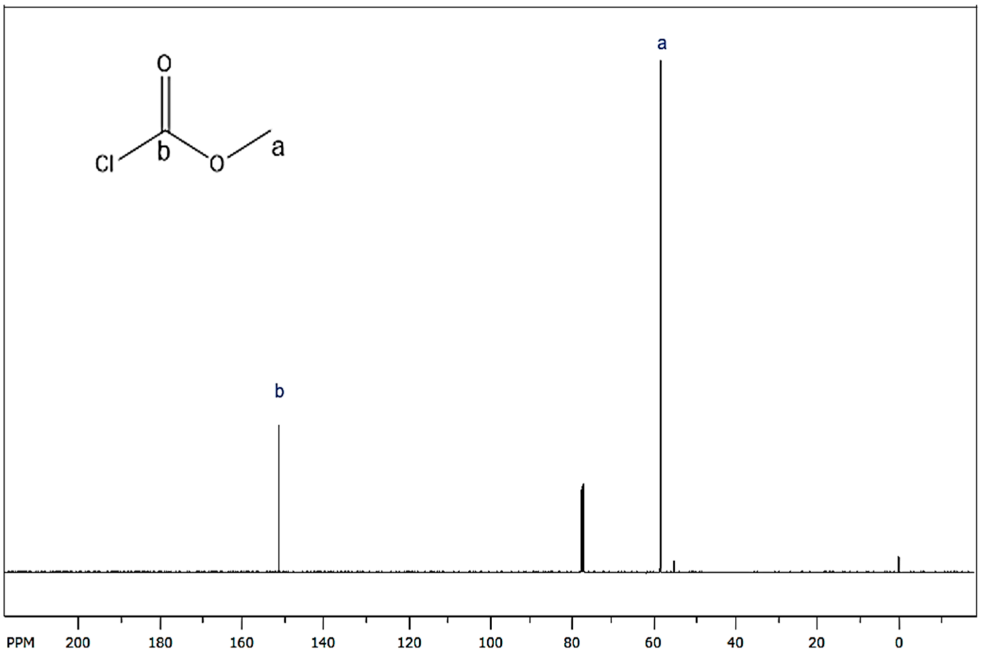

Figure S4. ^{13}C NMR spectrum of Methyl Chloroformate (500 MHz in $CDCl_3$ at room temperature, internal reference TMS).

The possitions of the atoms of the molecule methyl chloroformate (compound (**2**)) in Cartesian coordinates are shown in **Table S4**.

Table S4. Cartesian coordinates of methyl chloroformate (compound (2)).

Atomic Center	X	Y	Z
Cl	-2.826146	0.453305	0
O	-0.489893	-0.542639	0
O	-0.522298	1.720469	0
C	0.967608	-0.555898	0
C	-1.041171	0.651321	0
H	1.23307	-1.608051	0
H	1.339515	-0.059303	0.89423
H	1.339515	-0.059303	-0.89423

1.3. (R)-methyl-(2-hydroxy-1-phenylethyl)carbamate

The ^{1}H NMR spectrum obtained from the crude reaction is shown in **Figure S5**. This spectrum was useful for identifying the signals corresponding to the carbamat.

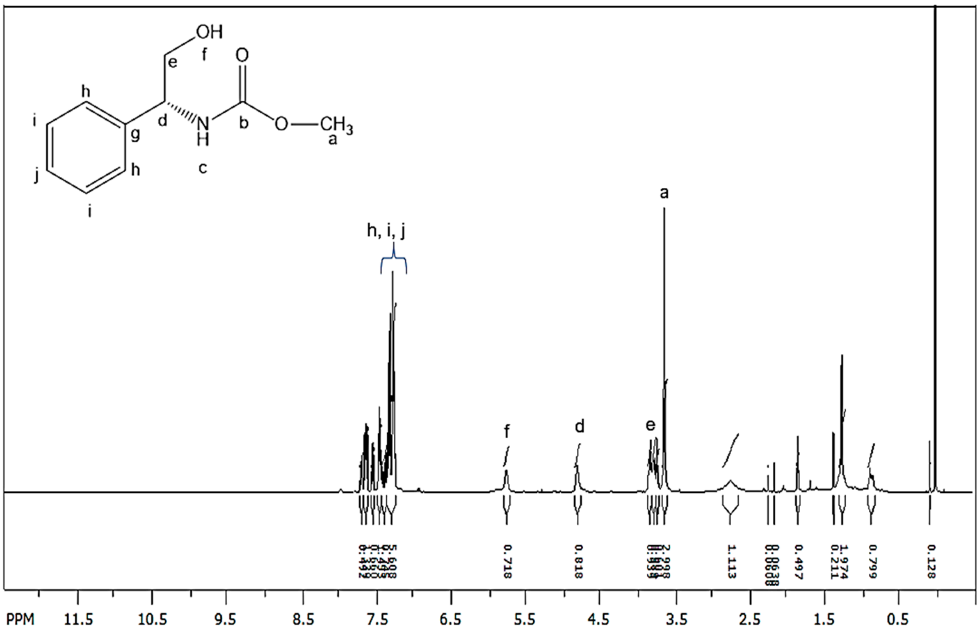

Figure S5. ^{1}H NMR spectrum of the crude reaction product 8 (500 MHz in $CDCl_3$ at room temperature, internal reference TMS).

In the spectrum, a singlet signal appears at 3.67 ppm, attributed to the hydrogens of the methoxy group (O-CH_3) (Ha), integrating for three protons. A doublet is observed at 3.87 ppm, corresponding to the diastereotopic hydrogens of the –CH_2 group (He) bonded to the -OH group, integrating for two protons. Additionally, two broad signals appear at 4.83 ppm and 5.5 ppm, corresponding to Hd and Hf, each integrating for one proton. These signals are associated with the hydrogen of the chiral carbon and the hydroxyl group (-OHf), respectively. In the aromatic region, a multiplet is observed between 7.29 and 7.38 ppm, attributed to the ortho, meta, and para hydrogens (Hh, Hi, Hj) of the phenyl ring, integrating for

five protons. This signal is composed of a collapsed doublet caused by the coupling between ortho hydrogens (Hh) and meta hydrogens (Hj), a doublet of doublets generated by the interaction between meta hydrogens (Hi) with ortho (Hh) and para (Hj) hydrogens, and a triplet produced by the coupling of the para hydrogen (Hj) with the two meta hydrogens (Hm). The summarized chemical shifts of the signals are presented in **Table S5**.

Table S5. Main signals of the ^{1}H NMR spectrum of (R)-methyl-(2-hydroxy-1-phenylethyl)carbamate.

Signal	Chemical Shift (ppm)	Integral	Multiplicity
Ha	3.67	3	Singlet
Hd	4.83	1	Broad singlet
He	3.87	2	Doublet
Hf	5.50	1	Broad singlet
Hh,i, j	7.29-7.38	5	Multiplet

The ^{13}C NMR spectrum of (R)-methyl-(2-hydroxy-1-phenylethyl)carbamate is shown in Figure S6. In this spectrum, a signal appears at 52.35 ppm, which corresponds to the carbon of the methoxy group (O-CH_3) (Ca). The signal observed at 57.10 ppm is attributed to the chiral carbon (Cd), while the signal at 66.52 ppm corresponds to the carbon bonded to the hydroxyl group (Ce). In the aromatic region, signals are detected between 128.5 and 128.8 ppm, which are associated with the ortho and para carbons (Ch, j) of the phenyl ring. Additionally, a signal at 132.05 ppm is assigned to the meta carbons (Cg) of the aromatic ring. Two additional signals are observed at 132.15 ppm and 157.09 ppm, corresponding to the ipso carbon (Cg) and the carbonyl carbon (Cb), respectively.

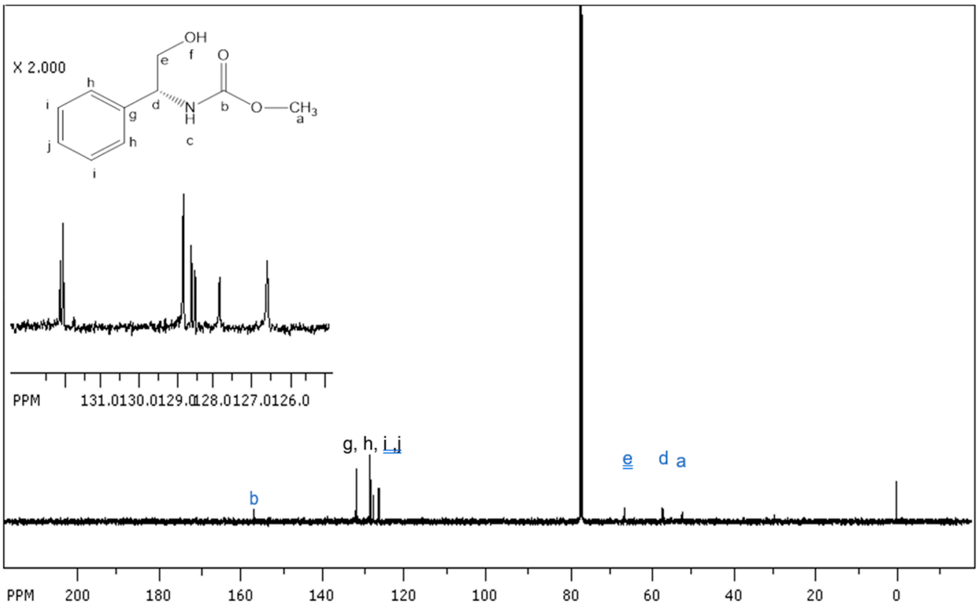

Figure S6. ^{13}C NMR spectrum of (R)-methyl-(2-hydroxy-1-phenylethyl)carbamate (500 MHz in $CDCl_3$ at room temperature, internal reference TMS).

These chemical shift assignments are consistent with previously reported data in the literature. The complete summary of the observed signals is provided in **Table S6**.

Table S6. Main signals of the ^{13}C NMR spectrum of (R)-methyl-(2-hydroxy-1-phenylethyl)carbamate

Signal	Chemical Shift (ppm)
Ca	52.35
Cb	157.09
Cd	57.10
Ce	66.52
Cg	132.15
Ch,j	128.5-128.8
Ci	132.05

The possitions of the atoms of (R)-methyl-(2-hydroxy-1-phenylethyl)carbamate (compound (**3**)) in Cartesian coordinates are shown in **Table S7**.

Table S7. Cartesian coordinates of (R)-methyl-(2-hydroxy-1-phenylethyl)carbamate (compound (**3**)).

Atomic Center	X	Y	Z
O	3.29648	-1.638919	-0.935518
N	2.439527	0.474637	0.553139
C	1.584803	-0.018025	-0.524509
C	0.100077	0.165533	-0.248382
C	1.892976	-1.504661	-0.719937
C	-0.76386	0.516558	-1.288403
C	-0.434783	-0.071968	1.021384
C	-2.136143	0.626565	-1.068546
C	-1.805381	0.040395	1.245419
C	-2.661214	0.388107	0.200328
H	1.833947	0.503298	-1.456065
H	1.319106	-1.872011	-1.577683
H	1.579968	-2.064682	0.170146
H	-0.359322	0.715972	-2.27665
H	0.228867	-0.329383	1.840205
H	3.235529	-0.106818	0.775237
H	-2.792607	0.906524	-1.886215
H	-2.205508	-0.141204	2.237937
H	-3.72831	0.47887	0.375671
H	3.52059	-2.576027	-0.930654
O	3.628834	2.033625	1.614502
C	3.883619	3.410058	1.93443
H	4.727966	3.396063	2.619495

H	4.135193	3.975808	1.036805
H	3.015198	3.862081	2.414849
O	1.922463	2.692661	0.263965
C	2.60133	1.813059	0.755094

1.4. 4-Phenyl-oxazolidine

From the reactions where the catalyst load was varied and the molar ratio was modified, a white solid identified as 4-phenyl-oxazolidine was successfully isolated and characterized through ^{1}H and ^{13}C NMR spectroscopy. In the ^{1}H NMR spectrum (Figure S7), a doublet of doublets is observed at 3.82 ppm, corresponding to the hydrogens of the $-CH_2$ group (Hd) bonded to oxygen, integrating for two protons. Another doublet of doublets appears at 4.36 ppm, attributed to the hydrogen of the chiral carbon, integrating for one proton. A singlet at 4.69 ppm corresponds to Ha, integrating for two protons, which are associated with the hydrogens of the carbon bonded to both nitrogen and oxygen. In the aromatic region, a multiplet is detected between 7.30 and 7.37 ppm, attributed to the ortho, meta, and para hydrogens (Hf, Hg, and Hh) of the phenyl ring, integrating for five protons. The signal corresponding to the hydrogen atom of the -NHb group is not observed, which may be due to the quadrupolar moment of nitrogen affecting the relaxation time.

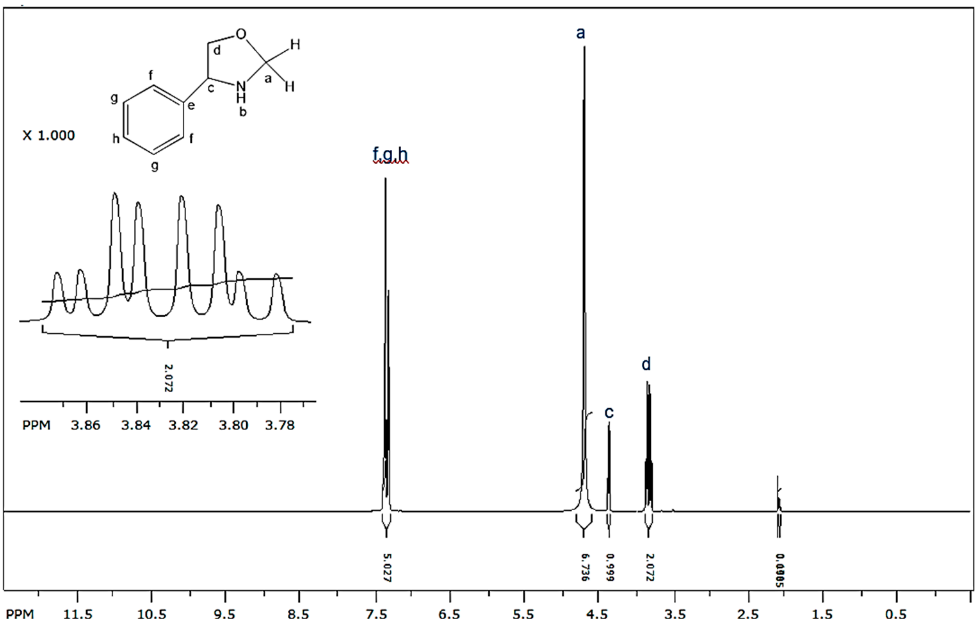

Figure S7. ^{1}H NMR spectrum of 4-phenyl-oxazolidine (500 MHz in $CDCl_3$ at room temperature, internal reference TMS).

It is important to note that the signal at 4.69 ppm shows an integration value of 7, which is higher than expected. This discrepancy is attributed to the overlapping of signals from the protons of the $-OCH_3$ group and the hydrogen (H) in the compound $[Pd(Cl)(H)_2(OCH_3)]$, which is considered an intermediate within the catalytic cycle.

The characteristic signals of 4-phenyl-oxazolidine are summarized in **Table S8**.

Table S8. Main signals of the ^{1}H NMR spectrum of 4-phenyl-oxazolidine

Signal	Chemical Shift (ppm)	Integral	Multiplicity
Ha	4.69	2	Broad singlet
Hc	4.36	1	Doublet of doublets
Hd	3.82	2	Doublet of doublets
Hf,g,h	7.30-7.37	5	Multiplet

The ^{13}C NMR spectrum of 4-phenyl-oxazolidine (**6**) is shown in Figure S8. The analysis of the spectrum reveals several characteristic signals. A signal at 56.29 ppm corresponds to the chiral carbon (Cc), while the signal at 62.42 ppm is attributed to the -CH_2 group (Cd) bonded to oxygen. A signal at 127.02 ppm is associated with the carbon (Ca) connected to both the amine (-NH) and the oxygen atom (O). The signals appearing between 129.22 and 129.50 ppm corres-pond to the ortho, meta, and para carbons (Cf, h, g) of the aromatic ring. Additio-nally, the signal at 133.50 ppm is assigned to the ipso carbon (Ce) of the aromatic ring.

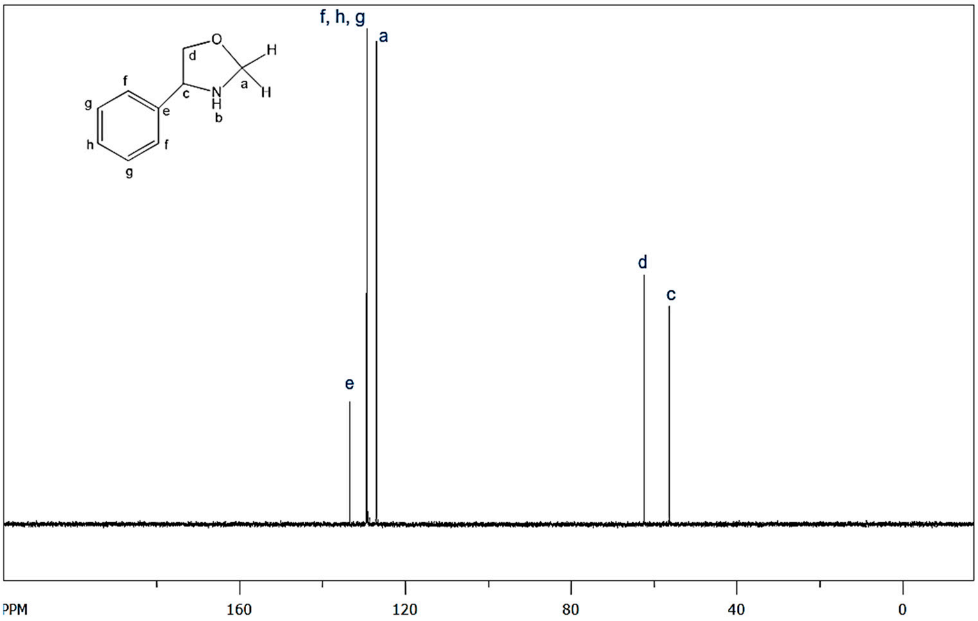

Figure S8. ^{13}C NMR spectrum of 4-phenyl-oxazolidine. (500 MHz in $CDCl_3$ at room temperature, internal reference TMS).

These chemical shift assignments are consistent with the expected structure of 4-phenyl-oxazolidine and provide essential evidence for structural characteri-zation. The summarized signals are presented in **Table S9**.

Table S9. Main signals of the ^{13}C NMR spectrum of 4-phenyl-oxazolidine.

Signal	Chemical Shift (ppm)

Ca	127.02
Cc	56.29
Cd	62.42
Ce	133.50
Cf, g, h	129.22-129.50

The possition of each atom of the molecule 4-phenyl-oxazolidine (compound (**6**)) in Cartesian coordinates is shown in **Table S10**.

Table S10. Cartesian coordinates of 4-Phenyl-oxazolidine (compound (**6**)).

Atomic Center	x	y	z
C	1.834485	1.631226	4.189313
C	1.755827	3.02912	4.241074
C	2.426625	0.954864	5.257498
C	2.264883	3.733383	5.329589
H	1.285434	3.57326	3.426084
C	2.938718	1.658137	6.34857
H	2.468373	-0.127602	5.230411
C	2.861573	3.048725	6.388882
H	2.191693	4.816169	5.353454
H	3.395061	1.11563	7.17086
H	3.257091	3.595164	7.239051
N	1.297984	-0.576435	3.090232
H	0.389569	-0.923707	2.804093
C	2.28707	-1.021935	2.12239
H	3.286532	-1.008457	2.57697
H	2.068074	-2.019122	1.742402
C	1.288991	0.901441	2.973692
H	0.260086	1.232272	2.810116
C	2.132596	1.172808	1.677158
H	1.649847	1.863829	0.985961
H	3.124459	1.561293	1.940691
O	2.249862	-0.092992	1.035994

The possitions of the atoms of the catalyst (compound (**4**)) and the intermediates species (compound (**7**), (**8**) and (**12**)) in Cartesian coordinates are shown in **Table S11, S12, S13** and **S14**, respectively.

Table S11. Cartesian coordinates of Tetratikis(trifenilfosfina)palladium ($Pd(PPh_3)_4$) (compound (**4**)).

Atomic Center	x	y	z
Pd	1.980248	0.035489	0.093148
P	-5.503797	-0.120364	0.148258

P	2.305322	-1.931402	-1.217896
P	1.945008	2.19873	-0.91703
P	1.687029	-0.140378	2.461896
C	0.825491	-3.050542	-1.352131
C	-0.059714	-3.103474	-0.26611
C	0.547842	-3.834249	-2.480507
C	-1.18423	-3.925965	-0.300911
H	0.135115	-2.495441	0.610222
C	-0.581794	-4.651601	-2.518898
H	1.212347	-3.808183	-3.336413
C	-1.450115	-4.701411	-1.42926
H	-1.855298	-3.954652	0.551464
H	-0.78058	-5.251358	-3.401609
H	-2.328966	-5.337339	-1.460031
C	2.806597	-1.794122	-3.007738
C	3.874632	-2.498953	-3.575989
C	2.078465	-0.916219	-3.823661
C	4.20237	-2.33286	-4.923105
H	4.45688	-3.184466	-2.971648
C	2.39387	-0.761633	-5.170158
H	1.261222	-0.341666	-3.400026
C	3.461747	-1.468478	-5.725067
H	5.036755	-2.885822	-5.343473
H	1.814223	-0.079271	-5.78349
H	3.716093	-1.340787	-6.772354
C	3.622505	-3.062757	-0.555915
C	3.511254	-4.457873	-0.525017
C	4.787128	-2.47164	-0.044147
C	4.538819	-5.241411	0.002303
H	2.61963	-4.94041	-0.908738
C	5.818962	-3.253429	0.469687
H	4.878565	-1.389676	-0.037554
C	5.695934	-4.643148	0.497388
H	4.432756	-6.321539	0.023613
H	6.713137	-2.777172	0.859075
H	6.494372	-5.25368	0.906568
C	-5.447066	-0.416494	-1.680647
C	-6.282233	0.214565	-2.611492
C	-4.474374	-1.312541	-2.149845
C	-6.15296	-0.049995	-3.975039
H	-7.035227	0.918079	-2.274225
C	-4.35357	-1.586292	-3.510613
H	-3.802347	-1.796877	-1.447318
C	-5.191905	-0.952537	-4.427758

H	-6.806629	0.449261	-4.683387
H	-3.596976	-2.284466	-3.853643
H	-5.0926	-1.156879	-5.488859
C	-6.680219	-1.425631	0.737856
C	-6.678843	-1.708163	2.112242
C	-7.534823	-2.15779	-0.096178
C	-7.524291	-2.680315	2.641926
H	-6.00869	-1.165308	2.772568
C	-8.372293	-3.140006	0.432292
H	-7.547054	-1.964955	-1.163057
C	-8.372302	-3.401206	1.801462
H	-7.51242	-2.882506	3.708154
H	-9.026425	-3.700377	-0.228279
H	-9.024425	-4.16592	2.210752
C	-6.512451	1.430151	0.276416
C	-5.829391	2.647558	0.127466
C	-7.88492	1.462124	0.552444
C	-6.503869	3.861647	0.231087
H	-4.761726	2.647101	-0.073165
C	-8.55801	2.678887	0.668568
H	-8.434096	0.536188	0.680302
C	-7.871688	3.880488	0.505316
H	-5.959254	4.792408	0.10848
H	-9.621636	2.684976	0.885221
H	-8.396826	4.825749	0.596156
C	0.274893	2.698262	-1.558769
C	-0.111028	4.032949	-1.748003
C	-0.645724	1.686219	-1.861789
C	-1.37774	4.343775	-2.23995
H	0.575597	4.836009	-1.504305
C	-1.911671	1.995077	-2.358259
H	-0.371092	0.650167	-1.690075
C	-2.279722	3.326065	-2.549867
H	-1.659644	5.382662	-2.379858
H	-2.61171	1.196937	-2.58267
H	-3.266356	3.568668	-2.931061
C	3.053083	2.410746	-2.395757
C	4.307412	1.786187	-2.351296
C	2.711195	3.140566	-3.540579
C	5.203898	1.902726	-3.410595
H	4.576729	1.192611	-1.482859
C	3.60188	3.245006	-4.609484
H	1.745371	3.627474	-3.608671
C	4.851359	2.631239	-4.546467

H	6.170591	1.412549	-3.355182
H	3.316446	3.808758	-5.492239
H	5.542795	2.713289	-5.378885
C	2.40111	3.697482	0.092442
C	3.481675	4.535694	-0.207372
C	1.652143	3.962149	1.250078
C	3.802677	5.611029	0.624294
H	4.078092	4.358641	-1.094426
C	1.962991	5.042417	2.070092
H	0.8218	3.315937	1.51522
C	3.043669	5.871005	1.76193
H	4.645576	6.247852	0.373974
H	1.366242	5.231745	2.956665
H	3.291332	6.708676	2.405945
C	1.290746	-1.789213	3.233504
C	0.165549	-2.025289	4.032571
C	2.150787	-2.863632	2.957592
C	-0.091259	-3.299224	4.544226
H	-0.517335	-1.216552	4.263746
C	1.904079	-4.128948	3.481551
H	3.017135	-2.711117	2.322463
C	0.777663	-4.353138	4.275199
H	-0.971348	-3.461549	5.158797
H	2.58611	-4.943589	3.260232
H	0.579255	-5.34214	4.67556
C	0.319791	0.916139	3.143888
C	0.40558	1.625577	4.347841
C	-0.861678	1.008277	2.393511
C	-0.663784	2.405086	4.79076
H	1.309563	1.577543	4.944135
C	-1.936122	1.772643	2.843557
H	-0.937107	0.484901	1.445236
C	-1.838118	2.477323	4.043705
H	-0.577391	2.953896	5.723419
H	-2.843966	1.824497	2.251243
H	-2.669921	3.08177	4.390841
C	3.161015	0.379586	3.466986
C	4.006118	1.362711	2.93331
C	3.470844	-0.160429	4.722866
C	5.123633	1.803232	3.640258
H	3.78452	1.782358	1.958208
C	4.593811	0.273984	5.426347
H	2.83829	-0.927481	5.154891
C	5.422277	1.257849	4.888237

H	5.763825	2.567519	3.211396
H	4.819837	-0.157563	6.396426
H	6.296485	1.594363	5.436346

Table S12. Cartesian coordinates of the compound (**7**), a central intermediate species for pathway 1 and 2.

Atomic Center	x	y	z
P	-0.563601	-1.747093	-0.042309
P	1.9331	1.103677	0.080045
C	-2.274328	-2.417013	0.180092
C	-2.846388	-2.347287	1.458069
C	-3.012287	-2.982169	-0.86268
C	-4.131036	-2.831773	1.685285
H	-2.286302	-1.920608	2.284548
C	-4.299256	-3.469964	-0.632136
H	-2.59157	-3.046598	-1.858871
C	-4.862554	-3.395037	0.63886
H	-4.560003	-2.771023	2.680221
H	-4.859666	-3.908	-1.45168
H	-5.863572	-3.774414	0.815798
C	-0.067291	-2.319222	-1.726375
C	0.683548	-3.473662	-1.975659
C	-0.469237	-1.523064	-2.810032
C	1.017546	-3.828746	-3.282358
H	1.027332	-4.093295	-1.157223
C	-0.142469	-1.886083	-4.114303
H	-1.035568	-0.615533	-2.630093
C	0.602924	-3.040138	-4.353607
H	1.604727	-4.723826	-3.459749
H	-0.463834	-1.261868	-4.941337
H	0.864125	-3.319281	-5.368991
C	0.342872	-2.789136	1.185446
C	0.129437	-4.175681	1.255892
C	1.189009	-2.191298	2.123438
C	0.774849	-4.942276	2.221335
H	-0.558003	-4.658574	0.570641
C	1.829373	-2.958723	3.096428
H	1.340217	-1.120107	2.10383
C	1.628975	-4.335312	3.143026
H	0.601674	-6.01262	2.26093
H	2.480618	-2.476411	3.817482
H	2.125736	-4.933405	3.899821
C	3.255041	-0.187133	0.155486

C	3.284396	-1.17437	-0.840342
C	4.21267	-0.230285	1.175294
C	4.25348	-2.173041	-0.820676
H	2.553003	-1.16335	-1.640629
C	5.178124	-1.237262	1.1976
H	4.2128	0.520885	1.955668
C	5.20331	-2.208726	0.200385
H	4.263528	-2.925252	-1.60243
H	5.913157	-1.255879	1.995709
H	5.956553	-2.989472	0.217443
C	2.363134	2.244136	1.465172
C	1.667393	2.133636	2.675064
C	3.379759	3.200141	1.351129
C	1.992931	2.950814	3.755658
H	0.854433	1.421616	2.766039
C	3.698284	4.023395	2.429709
H	3.920939	3.31167	0.418259
C	3.008683	3.897902	3.634838
H	1.442967	2.856629	4.686283
H	4.484017	4.764607	2.325932
H	3.256038	4.541101	4.473029
C	2.378695	2.054496	-1.447662
C	3.614658	1.910198	-2.091931
C	1.444683	2.968558	-1.958559
C	3.909081	2.660923	-3.229955
H	4.352745	1.21297	-1.714919
C	1.747696	3.717756	-3.093605
H	0.494722	3.091278	-1.446953
C	2.976693	3.564614	-3.734708
H	4.869966	2.537112	-3.719067
H	1.017609	4.422304	-3.479441
H	3.206969	4.146887	-4.621165
O	-0.663643	2.623556	0.257983
C	-5.251138	1.279756	0.450321
H	-4.869263	0.302113	0.7198
C	-4.390777	2.231574	-0.106846
C	-4.9386	3.4664	-0.489489
H	-4.298682	4.216336	-0.94553
C	-6.289846	3.746373	-0.305521
H	-6.687028	4.709796	-0.610819
C	-7.132726	2.790729	0.264326
H	-8.18703	3.005371	0.407993
C	-6.60736	1.556314	0.636456
H	-7.253963	0.798235	1.06841

N	-2.485726	0.589715	-0.041281
H	-2.792744	0.343261	0.902456
C	-2.013219	2.935062	0.508115
H	-2.271895	2.822101	1.578711
H	-2.191977	3.990123	0.244863
C	-2.899238	1.9827	-0.303411
H	-2.673419	2.18337	-1.360096
Pd	-0.436296	0.589523	0.113669

Table S13. Cartesian coordinates of the compound (**8**) an intermediate species for pathway 1.

Atomic Center	x	y	z
P	1.701102	1.751323	-0.013306
P	0.945323	-2.209252	-0.049953
C	0.850439	3.189829	0.775594
C	-0.453234	3.506999	0.373405
C	1.482928	4.000221	1.729329
C	-1.108447	4.615977	0.905503
H	-0.954545	2.892282	-0.362125
C	0.821995	5.103462	2.266521
H	2.490555	3.775215	2.057502
C	-0.473107	5.414732	1.854795
H	-2.115842	4.840463	0.572555
H	1.323502	5.720669	3.005051
H	-0.984415	6.275907	2.272904
C	3.097743	1.42277	1.156922
C	4.439143	1.638269	0.816789
C	2.795359	0.937715	2.437601
C	5.454125	1.372214	1.736634
H	4.698977	2.0191	-0.163269
C	3.809047	0.683549	3.356959
H	1.764869	0.763087	2.724339
C	5.142758	0.896833	3.008072
H	6.488153	1.546349	1.457258
H	3.555288	0.31266	4.344109
H	5.932736	0.694544	3.723789
C	2.550959	2.473985	-1.485673
C	2.722857	3.85117	-1.661957
C	3.046471	1.597835	-2.4609
C	3.382421	4.340643	-2.788962
H	2.33707	4.547362	-0.926722
C	3.714161	2.087482	-3.580455
H	2.908508	0.526916	-2.350206
C	3.881936	3.461748	-3.747773

H	3.502374	5.411514	-2.916446
H	4.091564	1.396141	-4.32667
H	4.392794	3.845084	-4.624901
C	2.558554	-2.728881	-0.7696
C	3.683759	-1.917926	-0.55735
C	2.690977	-3.889146	-1.541651
C	4.915515	-2.266194	-1.102661
H	3.607499	-1.020445	0.045347
C	3.926491	-4.229655	-2.091839
H	1.83847	-4.533865	-1.714416
C	5.039466	-3.421481	-1.874948
H	5.777707	-1.632507	-0.924666
H	4.014968	-5.131944	-2.68751
H	5.999393	-3.689577	-2.303258
C	-0.192541	-3.635561	-0.359344
C	-0.781374	-3.814065	-1.618607
C	-0.447342	-4.579135	0.643928
C	-1.600292	-4.91113	-1.870229
H	-0.604612	-3.097677	-2.413466
C	-1.277832	-5.670818	0.392777
H	-0.00773	-4.466853	1.626662
C	-1.854782	-5.840912	-0.862718
H	-2.046337	-5.031915	-2.851463
H	-1.472674	-6.385972	1.184872
H	-2.502937	-6.689134	-1.055428
C	1.177786	-2.229447	1.771441
C	2.21726	-2.945909	2.377336
C	0.22518	-1.578006	2.567363
C	2.301073	-3.010623	3.767032
H	2.959078	-3.455565	1.773952
C	0.313669	-1.653532	3.955731
H	-0.567034	-1.007185	2.090723
C	1.349208	-2.36846	4.557488
H	3.110194	-3.565754	4.229735
H	-0.425788	-1.147554	4.567701
H	1.415214	-2.424113	5.639093
O	-1.52853	-0.000248	0.437866
C	-5.759366	-0.495327	-1.206073
H	-5.27137	0.046258	-2.009522
C	-5.23005	-0.430091	0.086623
C	-5.882265	-1.119335	1.11353
H	-5.487487	-1.070698	2.124772
C	-7.037533	-1.856922	0.858739
H	-7.533981	-2.379965	1.67014

C	-7.558185	-1.914665	-0.433203
H	-8.460175	-2.483857	-0.634115
C	-6.915422	-1.229707	-1.464365
H	-7.316205	-1.265076	-2.472622
C	-2.724543	-0.622709	0.098357
H	-2.756564	-0.886075	-0.968197
H	-2.884515	-1.555348	0.662687
C	-3.933314	0.310272	0.376489
H	-3.912557	0.573102	1.438187
Pd	0.19433	-0.16342	-0.94931
C	-4.353105	2.717443	-0.156422
C	-6.010387	3.810707	1.125841
H	-6.762242	3.518154	1.855127
H	-5.33578	4.551015	1.557774
H	-6.48648	4.229359	0.239122
O	-5.304166	2.604122	0.80296
Cl	-0.810409	1.373041	-2.581082
O	-4.080806	3.768546	-0.714347
N	-3.761964	1.525022	-0.417
H	-2.950937	1.575595	-1.021842
H	-0.574491	-1.194933	-1.790244

Table S14. Cartesian coordinates of the compound (**12**) an intermediate species for pathway 2.

Atomic center	x	y	z
P	2.220293	0.885333	-0.491137
P	0.31618	-2.241929	0.32178
C	2.458934	2.700598	-0.774489
C	2.058492	3.252385	-2.002013
C	3.004611	3.543922	0.198976
C	2.208167	4.613439	-2.248749
H	1.641271	2.616748	-2.776318
C	3.155695	4.908319	-0.053234
H	3.328916	3.142541	1.151501
C	2.758505	5.446064	-1.273955
H	1.89421	5.023177	-3.202915
H	3.587757	5.547857	0.70939
H	2.875513	6.50711	-1.466936
C	3.363849	0.532451	0.918073
C	4.721565	0.22961	0.762278
C	2.829505	0.598396	2.212892
C	5.526562	0.00384	1.877453

H	5.157355	0.147173	-0.225036
C	3.637551	0.379622	3.326883
H	1.774636	0.810931	2.351146
C	4.988967	0.081168	3.161236
H	6.575588	-0.236648	1.739878
H	3.207368	0.433958	4.321234
H	5.617985	-0.096415	4.027146
C	3.002512	0.209189	-2.02237
C	4.197825	0.74337	-2.531278
C	2.365418	-0.80993	-2.735891
C	4.751147	0.244228	-3.706474
H	4.687986	1.56774	-2.026441
C	2.916288	-1.30506	-3.917407
H	1.427372	-1.20979	-2.374571
C	4.112575	-0.783561	-4.401368
H	5.675923	0.666373	-4.085518
H	2.405139	-2.094073	-4.458438
H	4.541764	-1.166245	-5.321416
C	1.854439	-3.173849	-0.142388
C	3.042501	-2.926497	0.561338
C	1.881431	-4.097419	-1.194963
C	4.220124	-3.588309	0.224975
H	3.050923	-2.223649	1.385882
C	3.063679	-4.755377	-1.534535
H	0.979442	-4.315109	-1.753414
C	4.235688	-4.504842	-0.826068
H	5.126265	-3.385985	0.786274
H	3.062283	-5.469325	-2.351872
H	5.154127	-5.01962	-1.088463
C	-0.987035	-3.160172	-0.609482
C	-1.346702	-2.710548	-1.885862
C	-1.581925	-4.324838	-0.110683
C	-2.265136	-3.420777	-2.656618
H	-0.917734	-1.794652	-2.278069
C	-2.509844	-5.0282	-0.877226
H	-1.327495	-4.686476	0.878802
C	-2.848864	-4.582276	-2.153953
H	-2.534043	-3.057153	-3.642805
H	-2.966318	-5.926858	-0.474931
H	-3.569386	-5.13217	-2.750415
C	0.088677	-2.738148	2.093377
C	0.673205	-3.897972	2.621914
C	-0.697642	-1.933786	2.930111
C	0.481537	-4.241237	3.959684

H	1.285264	-4.537135	1.997218
C	-0.885181	-2.281141	4.266834
H	-1.187527	-1.063765	2.511151
C	-0.294551	-3.431947	4.786875
H	0.941463	-5.142352	4.352399
H	-1.497514	-1.649402	4.902653
H	-0.440192	-3.698182	5.828854
C	-2.053282	4.639065	-0.39401
H	-2.454839	3.805858	-0.957317
C	-1.115656	4.382161	0.612419
C	-0.598706	5.469087	1.327593
H	0.139484	5.292591	2.105701
C	-1.008967	6.773669	1.055827
H	-0.592	7.600386	1.623388
C	-1.944248	7.015995	0.050588
H	-2.263223	8.030434	-0.168267
C	-2.462015	5.942362	-0.673652
H	-3.191615	6.119809	-1.458455
Pd	0.039784	0.171063	0.000694
C	-5.430153	-0.540105	-0.444409
C	-5.760121	-1.842667	-0.83869
C	-6.131365	0.525333	-1.021595
C	-6.759074	-2.077015	-1.781954
H	-5.227583	-2.682116	-0.400455
C	-7.131174	0.294838	-1.966574
H	-5.894266	1.53534	-0.708666
C	-7.449626	-1.006896	-2.351154
H	-7.002091	-3.095779	-2.068671
H	-7.663828	1.135221	-2.401743
H	-8.230583	-1.186739	-3.083463
C	-0.669664	2.958526	0.959648
H	0.338192	3.063738	1.404626
C	-1.526278	2.385419	2.100127
H	-1.40298	3.024349	2.989214
H	-1.154879	1.385372	2.346265
C	-2.958405	-0.095843	-0.212105
H	-2.915672	0.939383	-0.570987
H	-2.992168	-0.756798	-1.094642
C	-4.30215	-0.302721	0.546948
H	-4.168189	-1.225066	1.13555
N	-4.649604	0.83405	1.413007
H	-5.524975	0.649624	1.888558
N	-0.674896	2.096724	-0.226487
H	-0.082994	2.574155	-0.902203

O	-2.884324	2.318623	1.720495
O	-1.870986	-0.409268	0.60457
C	-3.659464	1.303012	2.345727
H	-2.985238	0.509028	2.691478
H	-4.185417	1.722771	3.214619
